# AGO2-RIP-Seq reveals miR-34/miR-449 cluster targetome in sinonasal cancers

**DOI:** 10.1371/journal.pone.0295997

**Published:** 2024-01-12

**Authors:** Marco Tomasetti, Federica Monaco, Corrado Rubini, Marzia Rossato, Concetta De Quattro, Cristina Beltrami, Giacomo Sollini, Ernesto Pasquini, Monica Amati, Gaia Goteri, Lory Santarelli, Massimo Re

**Affiliations:** 1 Department of Clinical and Molecular Sciences, Polytechnic University of Marche, Ancona, Italy; 2 Department of Excellence SBSP-Biomedical Sciences and Public Health, Polytechnic University of Marche, Ancona, Italy; 3 Department of Biotechnology, University of Verona, Verona, Italy; 4 ENT Division “Bellaria Hospital”, AUSL Bologna, Bologna, Italy; Concordia University, CANADA

## Abstract

Sinonasal tumours are heterogeneous malignancies, presenting different histological features and clinical behaviour. Many studies emphasize the role of specific miRNA in the development and progression of cancer, and their expression profiles could be used as prognostic biomarkers to predict the survival. Recently, using the next-generation sequencing (NGS)-based miRNome analysis the miR-34/miR-449 cluster was identified as miRNA superfamily involved in the pathogenesis of sinonasal cancers (SNCs). In the present study, we established an Argonaute-2 (AGO2): mRNA immunoprecipitation followed by high-throughput sequencing to analyse the regulatory role of miR-34/miR-449 in SNCs. Using this approach, we identified direct target genes (targetome), which were involved in regulation of RNA-DNA metabolic, transcript and epigenetic processes. In particular, the *STK3*, *C9orf78* and *STRN3* genes were the direct targets of both miR-34c and miR-449a, and their regulation are predictive of tumour progression. This study provides the first evidence that miR-34/miR-449 and their targets are deregulated in SNCs and could be proposed as valuable prognostic biomarkers.

## Introduction

Malignant tumours of the nasal cavity and paranasal sinuses (sinonasal cancers, SNCs) account for 0.2% of all human primary malignant neoplasms, with an incidence of 0.1–1.4 new cases/year/100,000 inhabitants [1]. SNCs are a heterogeneous group of rare tumours mainly associated with occupational exposure [2, 3]. Epithelial tumours, which include sinonasal adenocarcinoma (SNADC) and sinonasal squamous cell carcinoma (SNSCC), are linked to wood and leather dust exposure at the workplace [3, 4]. In addition, textile dusts, formaldehyde and organic solvents were also associated with epithelial SNC development [2]. SNSCC represents the most common subtype, followed by SNADC, which account for 50% and 20% of SNCs, respectively. Among the SNADCs, the intestinal-type sinonasal adenocarcinoma (ITAC) is more representative, with an incidence per 100,000 inhabitant-years ranging between 0.7 and 1.5 cases [5, 6]. Whereas sinonasal adenoid cystic carcinoma (SNACC), originating in the nasal cavity and paranasal sinuses, is responsible for 5–15% of sinonasal malignancies [7]. Other subtypes include sinonasal neuroendocrine carcinoma (SNEC), sinonasal undifferentiated carcinoma (SNUC) and sinonasal melanoma.

SNCs are aggressive tumours that grow silently with non-specific symptoms, which leads to late diagnosis, and are characterized by frequent local recurrence and a low survival rate [6, 8, 9]. Histopathological grading and TNM classification appear to be significant prognostic indicators [10].

Most of these tumours present at an advanced stage, and patients with SNCs show extensive tumour invasion of the surrounding tissue at diagnosis. Surgical resection with negative margins using an open or endoscopic approach is the gold standard treatment. Postoperative radiotherapy is also usually recommended to reduce the probability of local recurrence. Multimodal therapy, including chemotherapy, is also used depending on the tumour subtype, stage and experience of individual centres [11]. Anyway, the management of SNCs remain challenging due to their proximity to critical structures and location at the intracranial-extracranial interface, and radical surgery is associated, in many cases, with high morbidity, significant disfigurement, and adverse functional outcomes [12]. Therefore, since conventional clinical pathological parameters do not accurately reflect the clinical outcome of patients, the identification of early diagnostic biomarkers and prognostic biomarkers would be able to predict the outcome in patients affected by SNCs is essential. The pathogenesis of SNCs is not fully known, despite increasing information about the molecular events involved in the pathogenesis of head and neck squamous cell carcinomas (HNSCCs) [13, 14].

Recently, using NGS-based miRNome analysis, a miRNA expression profile has been identified and used as a promising non-invasive prognostic biomarker in ITAC [15]. Both miR‐34c, and miR‐449a were down-expressed in cancer tissue with respected to their non‐malignant counterparts, and their upregulation in cancer tissue significantly correlated with worse disease-free survival (DFS) and worse overall survival (OS). MiR-34 has low expression in many tumours, and plays an important role in tumour diagnosis, prognosis, treatment, and drug resistance [16]. In particular, the cluster miR-34/miR-449 was found to be involved in the molecular pathogenesis of SNC. The miR-449a was upregulated in sinonasal inverted papilloma (SNIP) and gradually was reduced during cell transformation from dysplasia to SNSCC malignant tissues [17]. Two main pathways are involved in the regulation of miR-34/449 expression. The tumour suppressor protein P53 promotes miR-34 expression in cancer, whereas the E2 promoter binding factor 1 (E2F1) directly transactivates miR-449a/b in cells. This differential regulation leads to distinct cellular phenotypes in cancer, highlighted that miR-34 primarily induces cell cycle arrest, while miR-449 induces apoptosis [16, 18].

In the present study, using AGO2: mRNA immunoprecipitation followed by high-throughput sequencing (AGO2-RIP-Seq), the miR-34/miR-449 targetome was identified and its direct targets proposed as potential prognostic biomarkers in SNCs.

## Materials and methods

### Ethics statement

The study was carried out according to the Helsinki Declaration and written informed consent was obtained from all participants. Ethical approval for this study (Ethical Committee N° 501) was provided by the Ethical Committee of the Marche Regional Hospital, Ancona, on 29 November 2011.

### Cell culture

Nasal septal squamous cell carcinoma (NSSCC) cells were obtained from the ATCC (RPMI 2650, CCL-30) and grown in RPMI-1640 with 10% FBS, 1% penicillin and 1% streptomycin in a humidified incubator at 37°C and in an atmosphere of 5% CO_2_. The cells were evaluated for the absence of mycoplasma contamination using the MycoAlert mycoplasma detection kit (item no. LT07-418, Lonza.com). The human cell line has been authenticated using STR profiling (PowerPlex Fusion 6C system, Promega, Fitchburg, WI, USA) within the last three years.

### AGO2-immunoprecipitation

AGO2-immunoprecipitation (IP) was performed as previously described [19, 20] and is summarized in the **S1 Fig**. NSSCCs (3.0 x 10^6^) were transiently transfected with a miR-34c or miR-449a mimic or scrambled control (100 nM, MISSION microRNA Mimic, Sigma, Merk, MI, Italy) using High Perfect Transfection reagent (item no. 301704, Qiagen, MI, Italy) according to the manufacturer’s instructions. After 72 hours of incubation, the cells were incubated with a lysis buffer containing 20 mM Tris-HCl pH 7.5, 150 mM NaCl, 0.05% NP40, 1 mM NaF, 2 mM EDTA, 0.5 mM DTT, HALT protease inhibitor cocktail (Thermo Fisher Scientific, MA USA), and 10 U/mL RNAse out (Invitrogen, Thermo Fisher Scientific, MA USA), for 15 minutes on ice. The lysates were centrifuged at 16,000g for 10 minutes at 4°C and incubated for 180 minutes with Protein G Sepharose beads (Thermo Fisher Scientific, MA USA) coupled with anti-AGO2 antibody (10 μg, Invitrogen, Thermo Fisher Scientific, MA USA) or rat IgG isotype control (10 μg, Millipore, Merk, MI, Italy). The beads were washed twice with lysis buffer for 10 minutes at 4°C and once with PBS. Bound material was eluted from beads with 50 μL 0.1 M glycine (pH 2.3) for 15 minutes at room temperature. The eluates were immediately neutralized with an equal volume of 1 M tris-HCl (pH 8) and treated with proteinase K (20 U) for 10 minutes at 65°C. Prior to RNA extraction, samples were spiked with 25 pmol of synthetic cel-miR-39 (Life Technologies, Grand 23 Island, NY) used as an endogenous control for library preparation and sequencing depth. The total lysate was used as an input sample. The total RNA was extracted in AGO2-IP, IgG-IP, and input samples by Trizol reagent (Sigma, Merk, MI, Italy) using an RNeasy Mini Kit (item no. 74004, Qiagen, MI, Italy) according to the manufacturer’s instructions. IPs were performed in three independent biological replicates.

### Cell proliferation, migration, invasion, and colony-forming assays

#### Cell proliferation

NSSCC cells were silenced for STK3, C9orf78 and STRN3 by siRNA (Validated MISSION Pre-designed siRNA, Sigma, Merk, MI, Italy) using High Perfect Transfection reagent (item no. 301704, Qiagen, MI, Italy) according to the manufacturer’s instructions. Silenced NSSCC cells and their parental counterparts were seeded in a 96-wells plate (2x10^4^ cells) and after 72 h of incubation the cells were evaluated by crystal violet assay.

#### Migration and invasion

The migration and invasion ability of STK3, C9orf78, STRN3 silenced NSSCC cells, and their parental counterparts were assessed using Transwell assay. The cells (1×10^5^) were suspended in RPMI-1640 medium, 2% FBS and seeded in the upper chamber of 96-well Transwell chamber (pore size 8 μm, Corning, Thermo Fisher Scientific, MA USA), 200μl RPMI-1640 medium, 20% FBS were added to the lower chamber. After incubation for 48 hours at 37°C, the lower surface was immersed in 70% ethanol cold solution, fixed for 15 min at 4°C, and stained with crystal violet. For the invasion assay, 30μl Geltrex (1:2, GIBCO, Thermo Fisher Scientific, MA USA) was applied on the upper surface of membrane. The remaining steps of the invasion experiment were the same as migration assay. The cells on the low chamber were visualized by microscope (Zeiss, Axiocam MRC5; magnification 20×), photographed, and calculated.

#### Colony-forming assay

NSSCC-silenced cells and their parental counterparts were seeded in a 6-well plate at the concentration of 2x10^3^ cells/well. After two weeks of incubation the colonies of NSSCC cells were stained with crystal violet (Sigma Aldrich, USA), visualized, and counted.

### Western blot analysis

NSSCC miR-34c/miR-449a mimic transfected cells and NSSCC control cells were lysed in RIPA buffer containing Na_3_VO_4_ (1 mM) and protease inhibitors (1 μg/ml). Protein concentration was assessed by the Bradford assay. The lysates (10–20 μg of protein) were separated using 4–12% SDS-PAGE (Life Technologies, Thermo Fisher Scientific, MA USA) and transferred onto a nitrocellulose membrane (Trans-Blot® Turbo™ Transfer System, Bio-Rad, CA, USA). After blocking with 5% non-fat milk in PBS-Tween (0.1%), the membranes were incubated overnight at 4°C with primary antibodies against STK3 (Antibodies.com), C9orf78 (Sigma) and STRN3 (Antibodies.com). β-Actin-HRP (Antibodies.com) was used as a loading control. After incubation with HRP-conjugated secondary IgG (Cell Signalling), the blots were developed using ECL (Thermo Fisher Scientific, MA USA). The band intensities were visualized and quantified with UVITEC chemiluminescence (Alliance Q9, Cambridge, UK) using nine Alliance software (Cambridge, UK).

### Study population

Patients with SNCs who underwent to primary surgery were retrospectively and prospectively recruited at the Otorhinolaryngology unit of the Regional Hospital of the Polytechnic University of Marche, Ancona, Italy, and at the ENT Division of “Bellaria Hospital”–AUSL Bologna, Italy, between 2011 and 2017. The medical charts of all patients were reviewed, and the demographic and clinical information collected, such as age, gender, occupational exposure, site of tumour, stage, histotype, surgery, chemotherapy, disease-free survival (DFS), overall survival (OS) and follow-up period.

Inclusion criteria were primary tumour obtained by endoscopic resection with or without transnasal craniectomy (ERTC) or by cranio-endoscopic resection, complete clinical data, a minimum of three years follow-up for patients without recurrence. All the patients signed a written informed consent to process their personal anonymized data at the time of enrolment.

Overall, 80 patients met the inclusion criteria. All patients had undergone complete clinical examination and were staged by multiplanar CT and contrast enhanced magnetic resonance imaging (MRI) or contrast-enhanced CT whenever an MRI could not be obtained, and PET/CT in advanced-stage lesions. After an imaging evaluation, a biopsy was obtained and stored at -80°C.

Evaluation of tumour sites (T1–T4), nodal involvement (N0–N3), and clinicopathological stage were performed in accordance with the American Joint Committee on Cancer TNM classification of malignant tumours [10]. Treatment planning was discussed by the local multidisciplinary team based on a common management strategy. All patients were treated by endoscopic resection, with or without ERTC or by cranioendoscopic resection, based on the local extent of disease.

The diagnosis and histotype assessment were performed on 4–6 μm thick FFPE sections stained with haematoxylin and eosin according to the World Health Organization (WHO) classification [21]. The adjuvant RT was discussed with each patient by the multidisciplinary team by considering histology, advanced stage, poor differentiation, and presence of positive surgical margins. All patients were followed as per our institutional protocols, which included an endoscopic evaluation and MRI every 4 months in the first year, every 6 months until the fifth year, and yearly thereafter.

Paired samples from tumour tissues and adjacent normal tissues were obtained from each patient and immediately preserved in RNAlater solution (item no. AM7020 Ambion, Thermo Fisher Scientific, MA USA), frozen at -20°C, and subsequently stored at -80°C.

### Quantitative RT-PCR

Total RNA was extracted from cells (10^6^) and biopsy samples (30 mg) using the RNeasy Mini Kit (item no. 74004, Qiagen, MI, Italy) kit according to the manufacturer’s instructions. The concentration and purity of RNA were determined by Nanodrop 1000 spectrophotometer (Thermo Fisher Scientific, MA USA). The miR-34c-3p and miR-449a-5p cDNA was synthesized using the TaqMan®Advanced miRNA cDNA Synthesis Kit (item no. A25576; Life Technologies, Grand 23 Island, NY). Quantitative RT-PCR (qRT‐ PCR) was performed through the TaqMan Fast Advanced Master Mix (item no. 4444557, Life Technologies, Grand 23 Island, NY) using miR-99b as the ’housekeeping’ normalizing gene. The qRT‐ PCR assays were performed using the Mastercycler EP Realplex (Eppendorf, MI, Italy) at the following conditions: 95°C for 20 seconds, followed by 40 cycles of 95°C for 1 second and 60°C for 20 seconds, with a final step at 4°C.

The *STK3*, *C9orf78* and *STRN3* first-strand cDNA was synthesized using a High-Capacity cDNA Reverse Transcription Kit (Life Technologies). The qRT‐ PCR was performed using the followed primers: *STK3* (fw-GAAATAGGCTATAACTGTGTGG, rw-CCCTCATTGGATGTATATCAG); *C9orf78* (fw-CAAAGATAAGATCAGTGAGGAG; rw-CCTTTCCTCTTCTTTAGCTC) and *STRN3* (fw-GAAGAAACCAAAGACACAGAG; rw-ACTGTCTTAAAAGCTGTCTG). The genes were detected by SYBR Select Master Mix (Life Technologies) with GAPDH as a housekeeping gene (fw-TCCACTGGCGTCTTCACC; rw-GGCAGAGATGATGACCCTTTT). The data were analysed using the automatic cycle threshold (Ct) setting to assign the baseline and the threshold for the determination of the Ct. The samples were analysed in duplicate and a Ct value >35 were excluded. The results were expressed as a relative expression (2^-ΔCT^) or as a fold-change (2^-ΔΔCT^).

### RIP-sequencing analysis

#### Library generation

cDNA libraries were generated from RNAs of the input, AGO2-IP and IgG-IP fractions by using the SMARTer Stranded Total RNA Sample Prep Kit (Takara Clontech, Diatech Lab Line Srl, Italy). The RNA-seq libraries were sequenced on a NovaSeq6000 (Illumina, CA, USA) analysing on average 82.8 M of fragments in 150PE mode for each sample. RNA-sequencing data presented in this study have been deposited in the Gene Expression Omnibus (GEO) database with accession number GSE192596.

### Bioinformatics data analysis

Filtered reads were aligned to the human reference genome (hg38) using STAR aligner (version 2.7.6a) with the option alignEndsProtrude 3 ConcordantPair which allows allow protrusion of alignment ends and peOverlapNbasesMin 5 which allows the merging and mapping of overlapping paired-end reads to improve mapping accuracy for overlapping.

Read counts were calculated by HTSEQ-count with the modality intersection-strict (version 0.13.5). Statistics of sequenced, mapping and number of identified genes are reported in **S1 Table**.

The sample corresponding to the Input of Scramble 1 has been considered as outlier sample based on samples distribution in the PCA plots (**S2 Fig**). This sample has been excluded from the analysis. For each miRNA (miR-449a and miR-34c) three comparisons have been performed: AGO2-IP miR vs AGO2-IP Scramble, IgG-IP miR vs IgG-IP Scramble and Input miRNA vs Input Scramble. The differentially expressed genes (DEGs) with p-value < 0.05 and Log_2_ Fold Change > 0 identified in each comparison have been intersected to remove possible false positive due to unspecific RNA binding to sepharose beads and widespread secondary changes due to miRNA overexpression. DEGs that are specific of the comparison AGO2-IP miR vs AGO2-IP Scramble are considered corrected for the noise of RIP-seq experiment. Subsequently, considering that miRNA targets expression is decreased after miRNA expression, to identify potential direct targets we have intersected down-regulated DEGs (log_2_ Fold Change <0) in Input miRNA vs Input Scramble and DEGs upregulated only in the comparison Ago-IP miR vs Ago-IP Scramble. We considered as potential direct targets of miRNAs DEGs that are both down-regulated (log_2_ Fold Change <0) in Input miRNA vs Input Scramble and upregulated only in the comparison AGO2-IP miR vs AGO2-IP Scramble.

### Statistical analysis

Results are expressed as mean ± standard deviation or as median, quartile and confidence interval (CI). The categorical variables were reported as fractions or percentages and compared with the chi-square method. Group comparisons were performed using the two-tailed Student’s t-test and analysis of variance (ANOVA), followed by post hoc Tukey analysis (more than two groups). Survival analysis was applied to evaluate the cumulative probability of OS and DFS. OS was defined from the date of surgery to the time of death, while DFS was defined as the duration between the completion of treatment and the diagnosis of recurrence. The cumulative incidence function (CIF) of OS and DFS was estimated by the Kaplan-Meier method, and for each variable, the CIFs for different groups were compared using the log-rank test. The Cox proportional hazard model was performed in a multivariate analysis to assess the effect of prognostic factors (age, gender smoking, staging and recurrence) on OS and DFS. Insignificant prognostic factors were excluded from the model using backward elimination (Wald). Probability values < 0.05 were considered significant. All statistical analyses were performed using the SPSS statistical package (SPPS Inc. Chicago, IL).

## Results

### miR-34c/miR-449a expression

The expression of miR-34c and miR-449a was evaluated in patients with SNC. The enrolled population consisted of 80 patients, which had a locoregional disease without distant metastases and had undergone radical surgery with tumour-free resection margins, followed by postoperative radiotherapy and/or chemotherapy in selected cases. There were 61 (76%) males and 19 (24%) females, with a mean age of 66 (range 33–90). Exposure to wood/leather was documented in 51% of patients. The tumour localization was in the nasal cavity in 22 patients (28%), in the medial wall of the maxillary sinus and ethmoid sinus in 42 patients (52%), and in the frontal recess and ethmoid sinus in 16 patients (20%). No patients showed positive nodes upon diagnosis.

The distribution by histologic type was as follows: 51 patients (64%) had a SNADC (the majority was ITACs, n = 46), 15 patients (19%) had a SNSCC, 5 patients (6%) had a SNACC, 5 patients (6%) had a SNUC, and 4 patients (5%) had a SNEC. Endoscopic resection with or without ERTC was the most used method for treatment of the primary lesion (64 patients, 80%). A cranio-endoscopic resection was performed in 16 patients (20%). According the TNM classification the tumour staging was distributed as follows: stage I, pT1N0M0, n = 3 (4%); stage II, pT2N0M0, n = 16 (20%); stage III, pT3N0M0, n = 26 (32%); stage IVa, pT4aN0M0, n = 27 (34%); stage IVb, pT4bN0M0, n = 8 (10%).

Adjuvant radiotherapy on the primary was delivered to 36 out of 80 patients (45%). Follow-up information was available with a median of 41.9 months [95% CI: 28.5–55.3 months], and no patients were lost to follow-up. During the follow-up period, with a median of 24.7 months [95% CI: 16.6–32.9 months], 36 patients (45%) developed a local relapse. All patients with local recurrence were treated with a surgical re-intervention ± RT or RT, and 32 patients (40%) died from the disease, with the main cause of death being local recurrence and intracranial invasion. The overall survival medians were: 43.1 [27.3–58.9] months for ITAC, 22.5 [10.2–30.8] for SNSCC, 79.4 [36.2–122.7] months for SNACC, 52.3 [0.0–112.7] months for SNUC, 31.2 [0.6–61.8] months for SNEC, 42.9 [0.0–87.0] months for SNADC, p = 0.023. The clinicopathological features of the patients are summarized in **Table 1**.

**Table 1:** Clinical and pathological characteristics of patients with primary sinonasal cancer.

*Characteristic*	*Value*
**Age,** mean yr ± SD	66 ± 13
**Sex** No. (%) Male Female	61 (76) 19 (24)
**Smoking** No. (%) Yes No former	34 (42) 39 (49) 7 (9)
**Occupational exposure** Yes No **Agent** Wood Leather	41 (51) 39 (49) 18 (44) 23 (56)
**Type of Surgery** No. (%) ERTC CR **Adjuvant therapy** No. (%) None RT	64 (80) 16 (20) 44 (55) 36 (45)
**Histotype** No. (%)**SNADC** • ITAC • non-ITAC **SNSCC** **SNACC** **SNUC** **SNEC**	51 (64) 46 (58) 5 (6) 15 (19) 5 (6) 5 (6) 4 (5)
**TNM classification** No. (%) Stage I Stage II Stage III Stage IVa Stage IVb	3 (4) 16 (20) 26 (32) 27 (34) 8 (10)
**Relapse,** No. (%) No Yes **Status** NED DOD	44 (55) 36 (45) 48 (60) 32 (40)

Abbreviations: SNADC, sinonasal adenocarcinoma; ITAC, Intestinal-type adenocarcinoma; non-ITAC, non-intestinal-type adenocarcinoma; SNSCC, sinonasal squamous cell carcinoma; SNACC, sinonasal adenoid cystic carcinoma; SNUC, sinonasal undifferentiated carcinoma; SNEC, sinonasal neuroendocrine carcinoma; ERTC, endonasal endoscopic resection with or without transnasal craniectomy; CR, cranioendoscopic resection; RT, radiotherapy; NED, no evidence of disease; DOD, died of disease.

**Fig 1:**
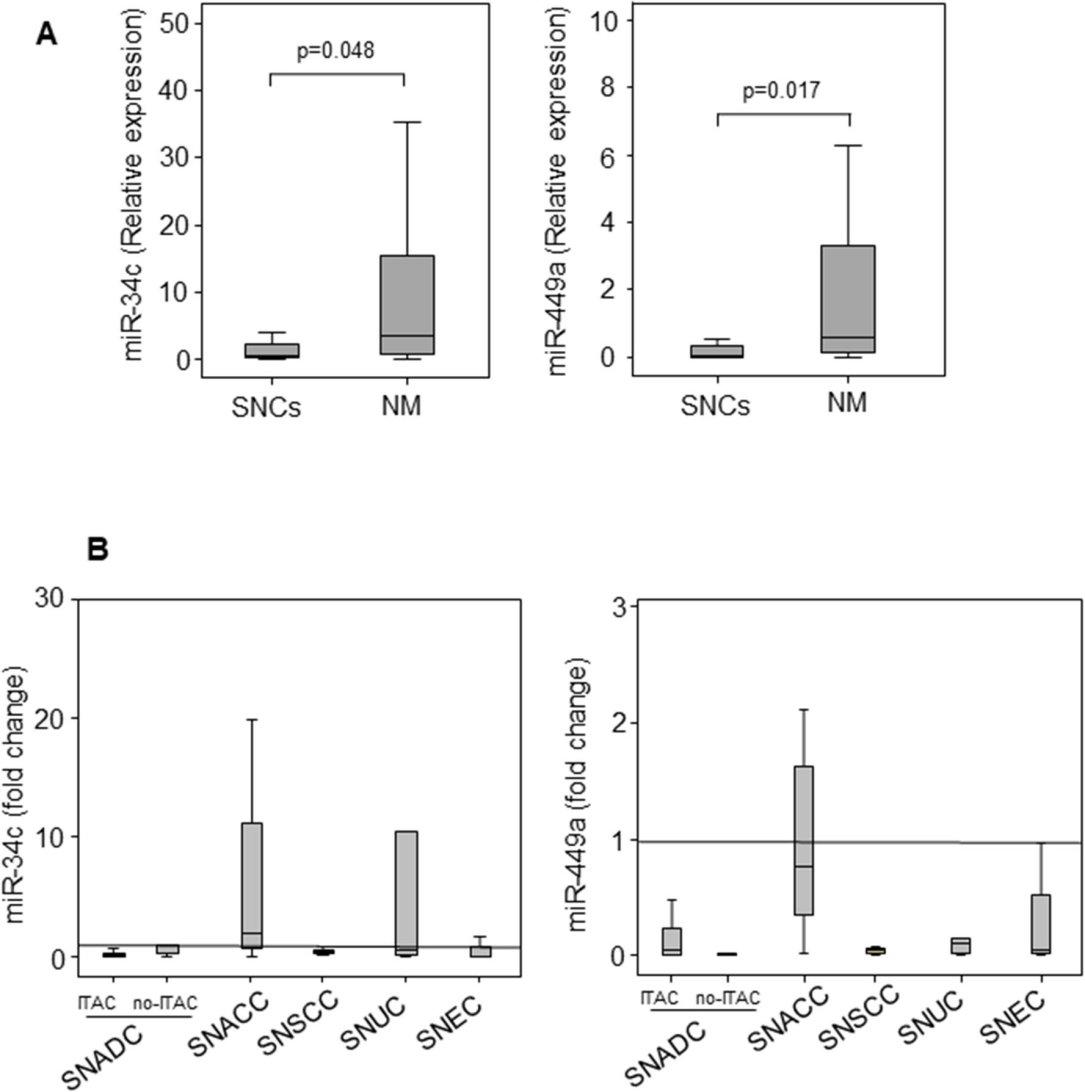
Distribution of miR-34c and miR-449a in SNCs. Expression of miR-34c and miR-449a in SNCs and non-malignant (NM) cells (**A**). Distribution of miR-34c and miR-449a in the SNC histotypes (**B**). Comparisons between and among groups were determined by Student’s t-test two-tailed (two groups) and by analysis of variance (ANOVA) followed by post-hoc Tukey analysis, respectively. Differences with p < 0.05 were considered statistically significant.

Results of qRT‐ PCR showed that both miRNAs were downregulated in SNCs when compared with the non-malignant (NM) counterparts, and were differently expressed in the different SNC histotypes, as shown in **Fig 1A and 1B**.

### Transcriptome identification

**Fig 2:**
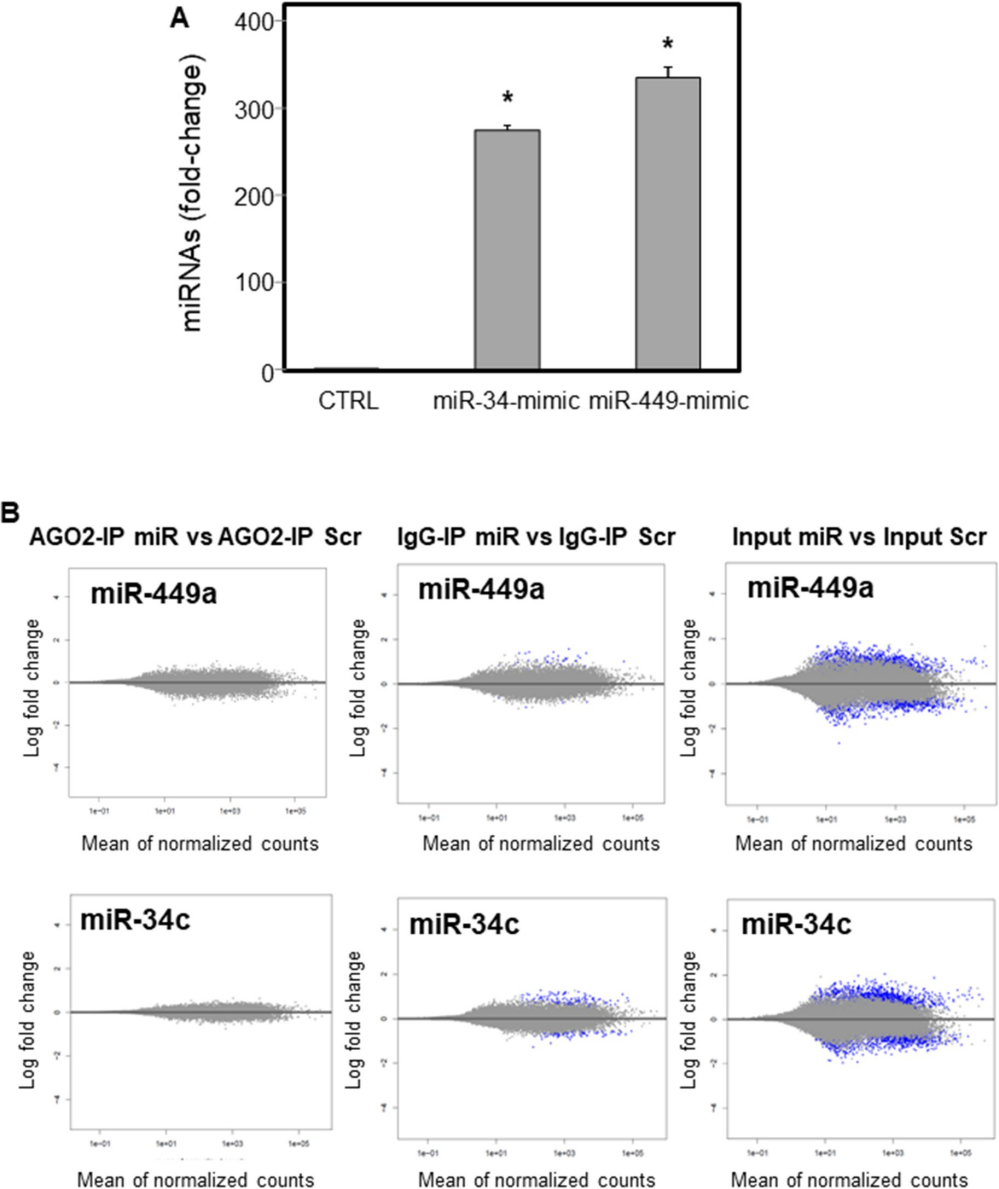
Analysis of miR-34c and miR-449a expression in NSSCC miR-34c/miR-449a mimic transfected cells and NSSCC control (empty vector transfection) cells. Relative miR-34c and miR-449a expression was adjusted to miR-99b (**A**). Comparison among groups was determined by analysis of variance (ANOVA), followed by post hoc Tukey analysis. Differences with p < 0.05 were considered statistically significant. * miR-34c mimic and miR-449a mimic vs. CTRL. The RNA extracts of the input and AGO2-IgG-IP fractions were analysed by high-throughput sequencing (AGO2-RIP-Seq) and comparisons between AGO2-IP miRNAs and AGO2-IP scramble, IgG-IP miRNAs and IgG-IP scramble and input miRNAs and input scramble were performed (**B**). The data were from three independent AGO2-IP experiments. MA plot of the three comparisons performed for both miR-449a and miR-34c.

To understand the oncogenic role of miR-34c/miR-449a in SNCs, we evaluated their direct target genes using a combination of AGO2-RIP and mRNA-Seq following overexpression of miR-34c/miR-449a in SNC cells. The miR-34c, miR-449a and miR-scramble (scr) as control were overexpressed in NSSCCs, they were lysed and the AGO2 complexes were immunoprecipitated from total lysate (input) with AGO2 antibodies (AGO2-IP) and with isotype IgG antibodies (IgG-IP) used as a control. As expected, miR-34c and miR-449a were enriched in NSSCCs and in the input fraction (300-400-fold compared to controls, **Fig 2A**).

The RNA extracts of the input and AGO2-IgG-IP fractions of three independent AGO2-IP experiments were analysed by high-throughput sequencing (AGO2-RIP-Seq). To statistically model the AGO2-RIP-Seq experiment, we defined the comparisons between AGO2-IP miRNAs and AGO2-IP scramble, IgG-IP miRNAs and IgG-IP scramble and input miRNAs and input scramble (**Fig 2B**). Next, differentially expressed genes (DEGs) in miR-34c and miR-449a treated groups were identified by comparing the mRNA expression in the AGO2-IP, IgG-IP and input groups. A total of 88 and 185 genes were specifically bound in the AGO2-complex of miR-34c and miR-449a, respectively. As target expression is known to be reduced by miRNAs, in parallel, we also compared these RNA profiles with transcriptome changes after miR-34c and miR-449a overexpression using RNA-Seq (comparison input). Potential miRNA targets were defined by positive log_2_FC values in AGO2-IP and negative log_2_FC in the input (total lysate). The integration of AGO2-RIP-Seq and RNA-Seq data yielded 29 potential direct targets for miR-34c and 47 direct targets for miR-449a (**S3 and S4 Figs**). The directly regulated target genes of miR-34c and miR-449a are listed in **Table 2**.

**Table 2:** Target genes.

miR-34c target gene	Gene description
DNAJC10	DnaJ heat shock protein family (Hsp40) member C10
NRDC	nardilysin convertase
URI1	URI1 prefoldin like chaperone
POLR1F	RNA polymerase I subunit F
DEK	DEK proto-oncogene
UBA2	ubiquitin like modifier activating enzyme 2
CCDC59	coiled-coil domain containing 59
CEP55	centrosomal protein 55
FRA10AC1	FRA10A associated CGG repeat 1
KIN	Kin17 DNA and RNA binding
GKAP1	G kinase anchoring protein 1
FAM204A	family with sequence similarity 204 member A
TPM4	tropomyosin 4
ARL13B	ADP ribosylation factor like GTPase 13B
CCDC43	coiled-coil domain containing 43
ZNF320	zinc finger protein 320
JRKL	JRK like
SEPTIN10	septin 10
ZNF568	zinc finger protein 568
RUFY2	RUN and FYVE domain containing 2
HMGB1P6	high mobility group box 1 pseudogene 6
AC245060.7	lncRNA
**miR-449a target gene**	**Gene description**
CSDE1	cold shock domain containing E1
ADSS2	adenylosuccinate synthase 2
EIF4B	eukaryotic translation initiation factor 4B
APLP2	amyloid beta precursor like protein 2
TXLNG	taxilin gamma
HSP90AB1	heat shock protein 90 alpha family class B member 1
ST13	ST13 Hsp70 interacting protein
RHOV	ras homolog family member V
GTPBP4	GTP binding protein 4
CCDC47	coiled-coil domain containing 47
CCDC34	coiled-coil domain containing 34
METAP2	methionyl aminopeptidase 2
CMAS	cytidine monophosphate N-acetylneuraminic acid synthetase
IFT57	intraflagellar transport 57
NCBP2	nuclear cap binding protein subunit 2
CACYBP	calcyclin binding protein
SET	SET nuclear proto-oncogene
ZCCHC9	zinc finger CCHC-type containing 9
UTP3	UTP3 small subunit processome component
HSD17B4	hydroxysteroid 17-beta dehydrogenase 4
CALCOCO2	calcium binding and coiled-coil domain 2
C1orf131	chromosome 1 open reading frame 131
TPBG	trophoblast glycoprotein
CCT6A	chaperonin containing TCP1 subunit 6A
EBAG9	estrogen receptor binding site associated antigen 9
CETN3	centrin 3
ZNF22-AS1	ZNF22 antisense RNA 1
UPF3A	UPF3A regulator of nonsense mediated mRNA decay
TAF7	TATA-box binding protein associated factor 7
ZFP3	ZFP3 zinc finger protein
NPM1	nucleophosmin 1
KATNA1	katanin catalytic subunit A1
GTF2F2	general transcription factor IIF subunit 2
FAM3C	FAM3 metabolism regulating signaling molecule C
NUDT16	nudix hydrolase 16
ZNF830	zinc finger protein 830
TYW1	tRNA-yW synthesizing protein 1 homolog
DHX16	DEAH-box helicase 16
GET1-SH3BGR	GET1-SH3BGR readthrough
**miR-34c/miR-449a target gene**	**Gene description**
**STK3**	serine/threonine kinase 3
PPWD1	peptidylprolyl isomerase domain and WD repeat containing 1
**C9orf78**	chromosome 9 open reading frame 78
ANP32B	acidic nuclear phosphoprotein 32 family member B
PRPF18	pre-mRNA processing factor 18
**STRN3**	striatin 3
L3MBTL3	L3MBTL histone methyl-lysine binding protein 3

Most of the genes were confirmed by using ‘in silico’ approaches (DIANA, miRanda, TargetScan), and were involved in regulation of RNA-DNA metabolic processes and ATP-histone binding, transcript and epigenetic regulation. Next, by using STING pathway analysis, the protein-protein interaction network of all targets specifically bound to the Ago-IP-miR-34c/miR-449a was evaluated. As shown in **S5 Fig**, interaction nodes are found for DNA-binding general transcription factor complex and nucleolar GTP-binding protein-3, involving the zinc finger proteins for heat shock protein HSP 90β, for Ras-related protein RAB involved in membrane trafficking between the Golgi complex and endosomes, and insulin-like growth factor-binding protein, which involves amyloid-like protein-2 regulations. Over the regulated targets, seven genes were co-regulated by both miR-34c and miR-449a (**Table 2**).

### Expression of STK3, C9orf78 and STRN3 in SNCs

The expression of *STK3*, *C9orf78* and *STRN3* (miR-targets) was evaluated in biopsies collected from 80 patients with SNCs.

None of the three miR-target genes was differentially expressed in tumours with respect to their non-malignant counterparts (median 151.2 [4.8–41932.6] vs. 193.4 [14.8–4958.8], p = 0.141 for *STK3*; median 70.8 [2.4–2128.7] vs. 89.4 [1.2–6453.1], p = 0.337 for *C9orf78*; and median 54.8 [1.0–11551.4] vs. 90.3 [8.1–19562.2], p = 0.986 for *STRK3*). Strong positive correlations were found among the three miR-targets (rho = 0.959, p < 0.0001 for *STK3* vs. *STRN3*; rho = 0.868, p < 0.0001 for *STK3* vs. *C9orf78*; and rho = 0.887, p < 0.0001 for *STRN3* vs. *C9orf78*), while no correlations were found with miR-34c and miR-449a.

**Fig 3:**
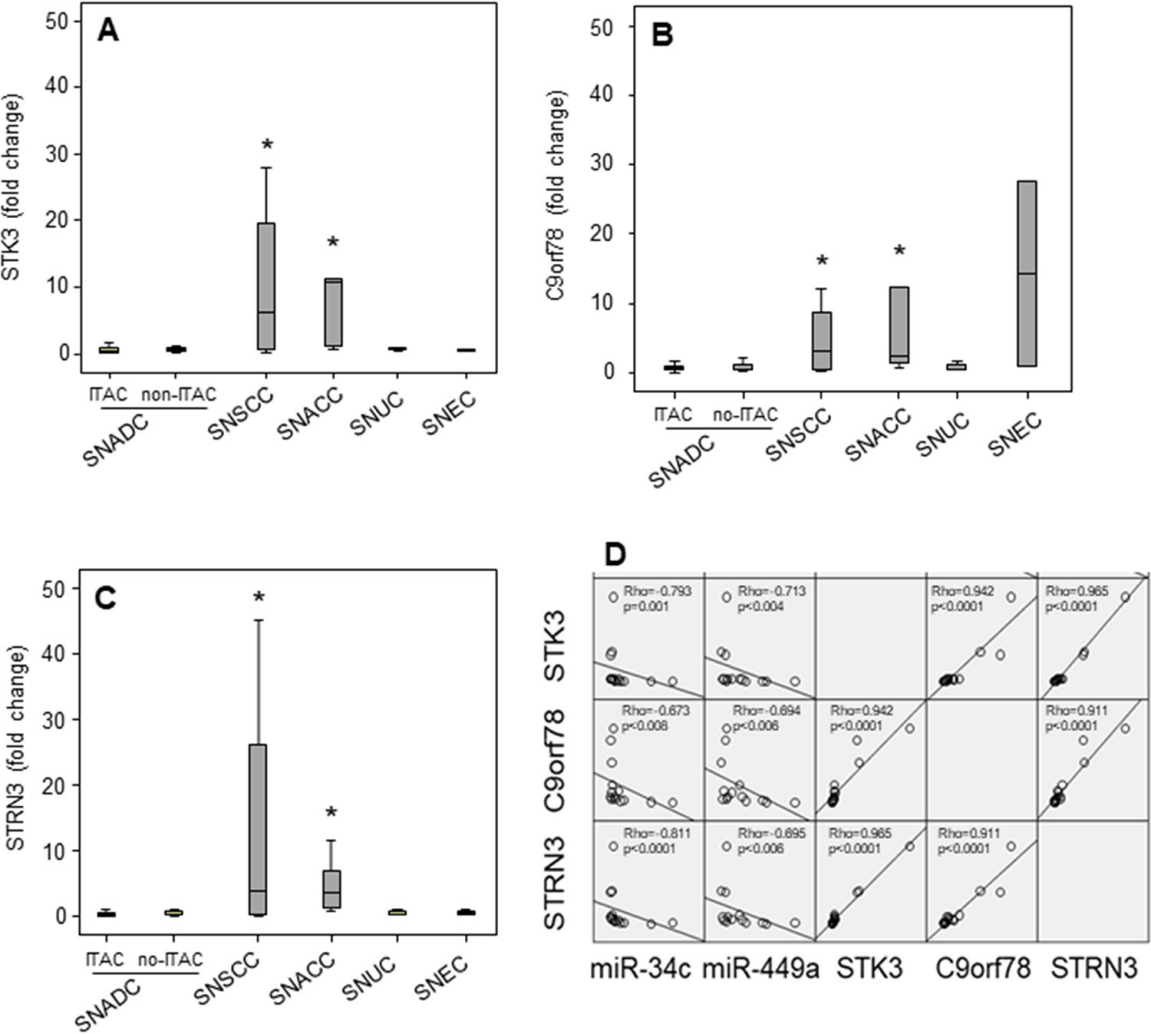
Distribution of STK3, C9orf78 and STRN3 gene expression in the SNC histotypes. Expression of STK3 (**A**), C9orf78 (**B**) and STRN3 (**C**) in tumour with respect to their non-malignant counterparts (fold-change) in the SNC histotypes. Spearman’s correlation among STK3, C9orf78 and STRN3 and miR-34c and miR-449a in the SNACC histotype (**D**). Comparison among groups was determined by analysis of variance (ANOVA), followed by post hoc Tukey analysis. Differences with p < 0.05 were considered statistically significant. * SNACC and SNSCC vs. SNADC, SNUC and SNEC.

By stratifying for the histotype, low expression (fold-change) of *STK3*, *C9orf78* and *STRN3* was found in SNADC (ITAC and non-ITAC), SNUC and SNEC, while a significant increase of protein expression was observed in SNSCC and SNACC. Within the SNACC histotype, both miR-34c and miR-449a negatively correlated with the *STK3*, *C9orf78* and *STRN3* (**Fig 3**).

### Correlation of the selected miR-target genes with patient outcome

To assess the clinical value of *STK3*, *C9orf78* and *STRN3*, their expression levels were correlated with the outcome of the patients with SNCs. The ITAC (n = 46, age 69.0 ± 13.6 yrs, male = 46) and SNSCC (n = 15, age 69.9 ± 5.2 yrs, male = 12 and female = 3) groups were selected as representative histotype with low and high expression of *STK3*, *C9orf78* and *STRN3*, respectively. OS and DFS were analysed by means of the Kaplan–Meier curve, and STK3, C9orf78 and STRN3 groups (below and above the median rate) were compared by log-rank test. Statistically significant variables were entered into a multivariate Cox regression model.

**Fig 4:**
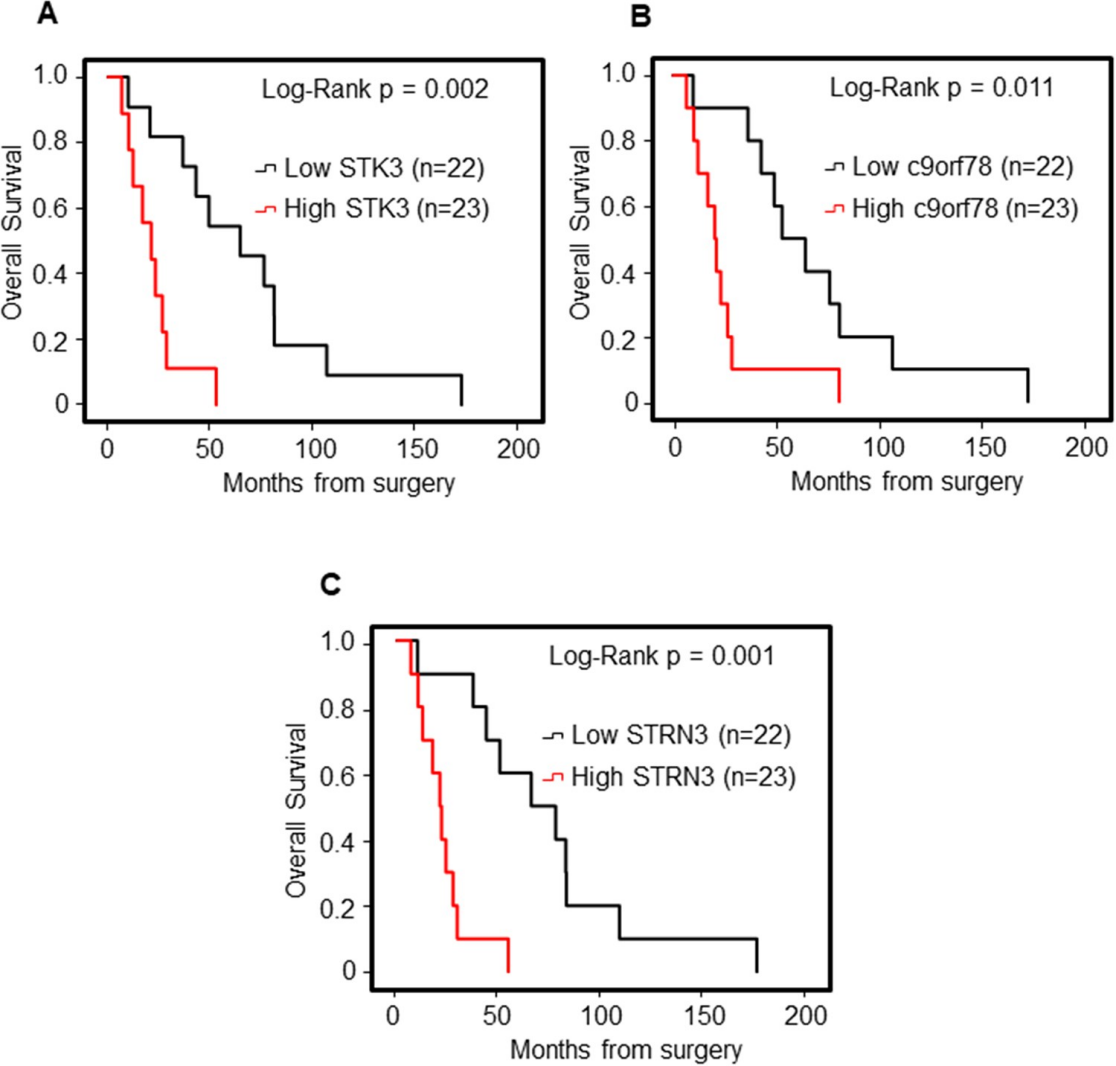
Kaplan-Meier survival curves for ITAC stratified for STK3, C9orf78 and STRN3 gene expression. Low and high gene expression of STK3 (**A**), C9orf78 (**B**), and STRN3 (**C**) were associated with overall survival (OS). Comparisons between groups were made using the log-rank test and p < 0.05 were considered statistically significant.

Within the group of patients with low *STK3*, *C9orf78* and *STRN3* expression (ITAC) the low level of miR-target genes was associated with significant better OS: *STK3*, median OS was 64.7 (95% CI: 28.7–100.7) months vs. 21.3 (95% CI: 7.5–9.6) months, p = 0.002; *C9orf78*, median OS was 53.4 (95% CI: 29.8–77.0) months vs. 20.5 (95% CI: 14.0–27.1) months, p = 0.011; *STRN3*, median OS was 64.7 (95% CI: 22.9–106.5) months vs. 20.5 (95% CI: 14.3–27.8) months, p = 0.001 (**Fig 4**).

At multivariate analysis, considering age, smoking status, staging, and recurrence, only *STRN3* in association with the stage and recurrence resulted independent prognostic factors associated with OS (**S2 Table**).

Conversely, within the group of patients with highly expressed *STK3*, *C9orf78* and *STRN3* (SNSCC) the low expression levels of the miR-target genes were associated with worse OS: *STK3*, median OS 79.4 (95% CI: 43.2–115.6) months vs. 11.6 (95% CI:10.0–13.6) months, p = 0.022; *C9orf78*, median OS 79.4 (95% CI: 13.8–145.0) months vs. 11.8 (95% CI: 0.0–45.5) months, p = 0.05; *STRN3*, median OS 79.4 (95% CI: 53.2–105.7) months vs. 11.6 (95% CI: 0.39–23.2) months, p = 0.007.

**Fig 5:**
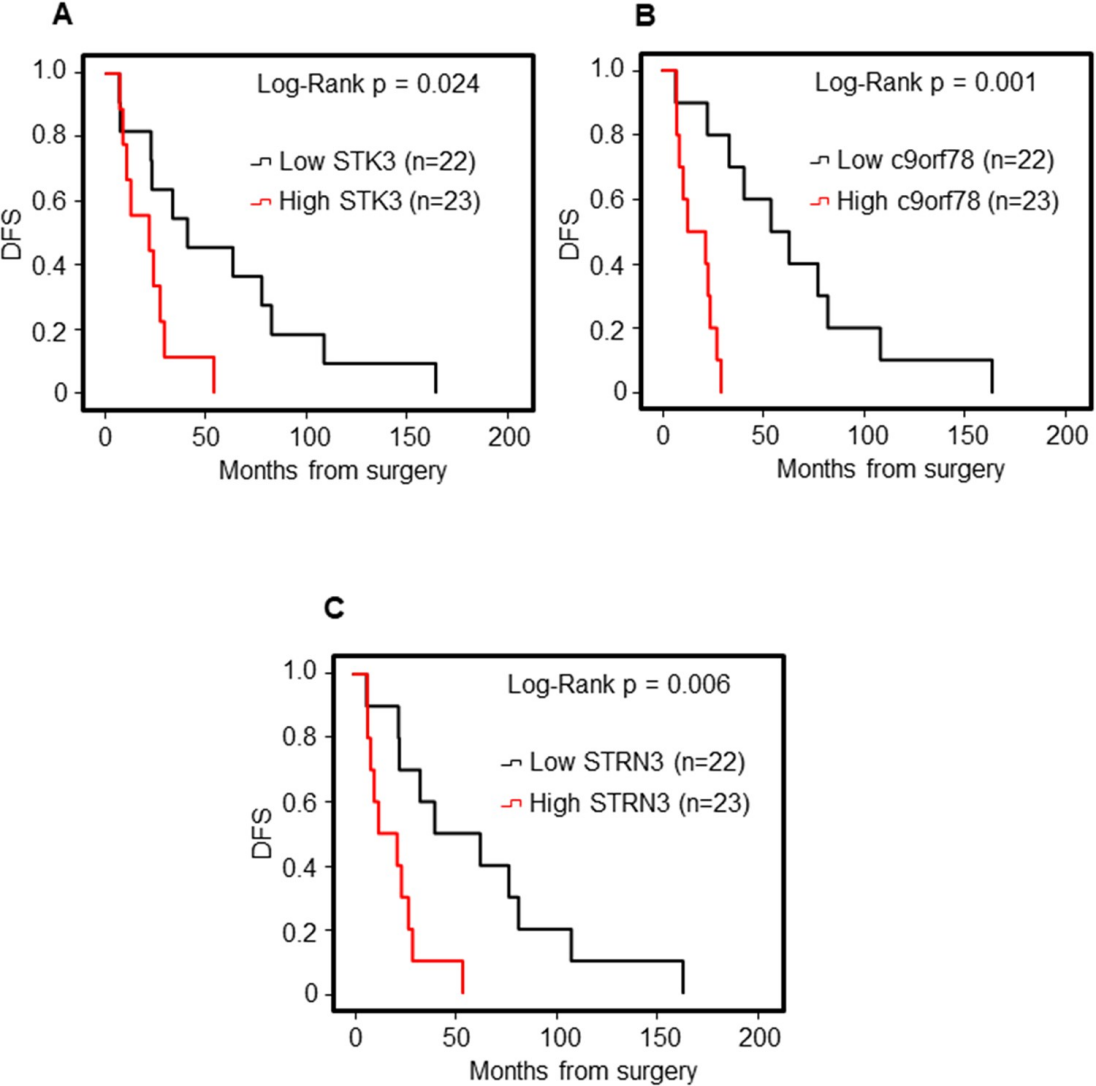
Kaplan-Meier survival curves for ITAC stratified for STK3, C9orf78 and STRN3 gene expression. Low and high gene expression of STK3 (**A**), C9orf78 (**B**), and STRN3 (**C**) were associated with disease-free survival (DFS). Comparisons between groups were made using the log-rank test and two-sided p < 0.05 were considered statistically significant.

The assessment of the disease-free survival (DFS) in the selected cohort using the same cut-off points showed the same trend. The *STK3*, *C9orf78* and *STRN3* were significant prognostic predictors for DFS through univariate analysis. Specifically, high expression levels of these miR-target genes were associated with a higher recurrence risk in ITAC, and better prognosis in SNSCC (**Fig 5**).

At multivariate analysis with Cox proportional hazard model only *STRN3* was confirmed as prognostic predictors for DFS (**S2 Table**).

### Role of miR-34/miR-449 target genes in tumorigenicity

**Fig 6:**
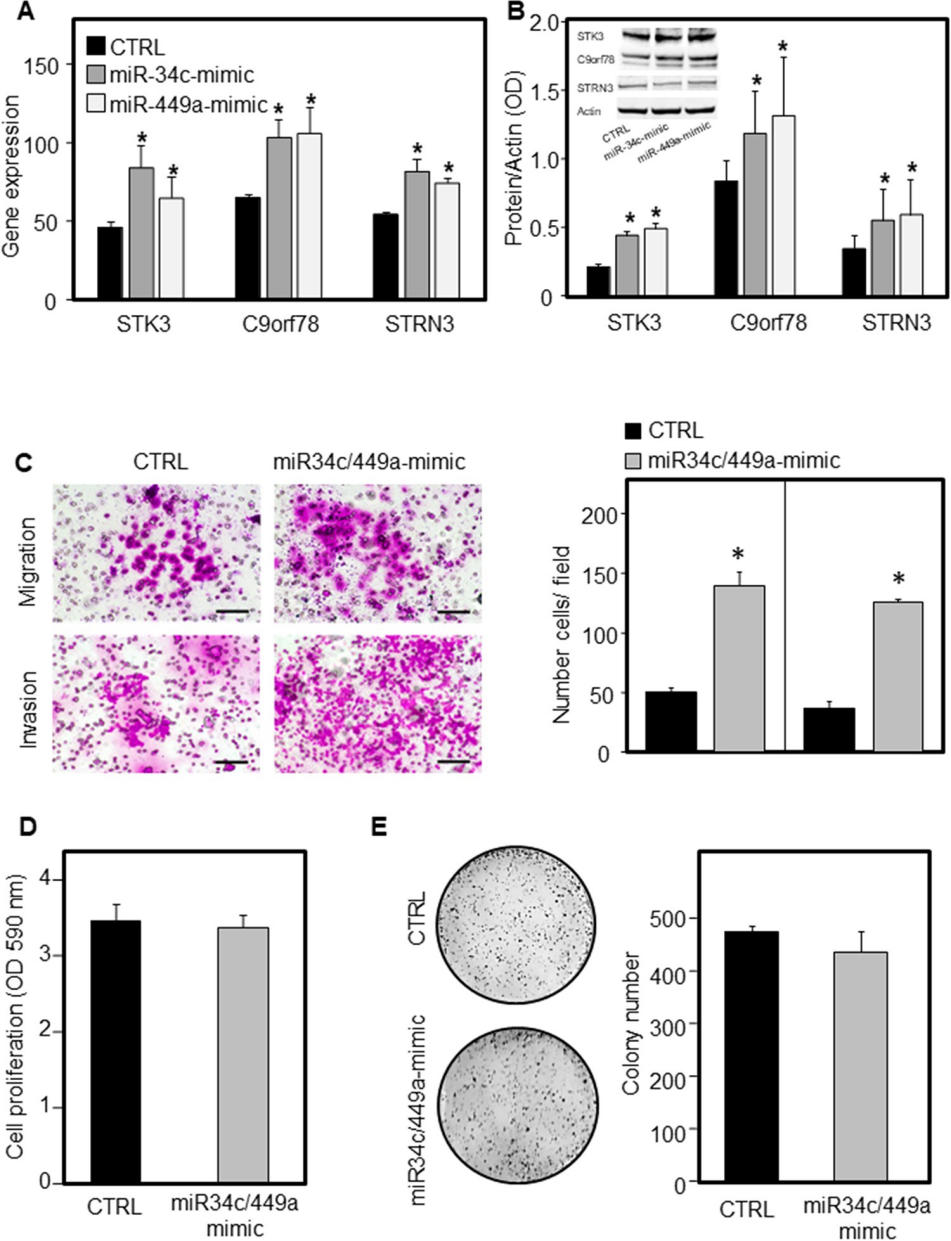
MiR-34/miR-449-induced STK3, C9orf78, STRN3 expression in NSSCC cells. NSSCC cells were transiently transfected with miR-34c/miR-449a mimic and evaluated for STK3, C9orf78 and STRN3 gene (**A**), and protein expression (**B**). The protein levels were evaluated by densitometry analysis of the bands relative to actin. The miR-34c/miR-449a-transfected NSSCC cells and their parental counterparts were investigated for cell migration/invasion (**C**), cell proliferation (**D**) and colony forming assay (**E**). Scale bar = 100 μm. Comparisons between and among groups were determined by two-tailed Student’s t-test and analysis of variance (ANOVA) followed by post-hoc Tukey analysis. Differences with p < 0.05 were considered statistically significant.

The role of miR-34/miR-449 direct target genes in the tumorigenicity was in vitro evaluated by gain- and loss-of-function experiments using nasal septal squamous cell carcinoma (NSSCC) cells. The STK3, C9orf78 and STRN3 overexpression was obtained by transfecting NSSCC cells with miR-34/miR-449-mimic. Ectopic miR-34/miR-449, rather than inhibiting, induced the expression of STK3, C9orf78 and STRN3 either as mRNA or protein (**Fig 6A and 6B**). The miR-34/miR-449-induced STK3, C9orf78 and STRN3 overexpression significantly enhanced both migration and invasion of NSSCC cells (**Fig 6C**), without affecting cell proliferation and colony formation (**Fig 6D and 6E**).

**Fig 7:**
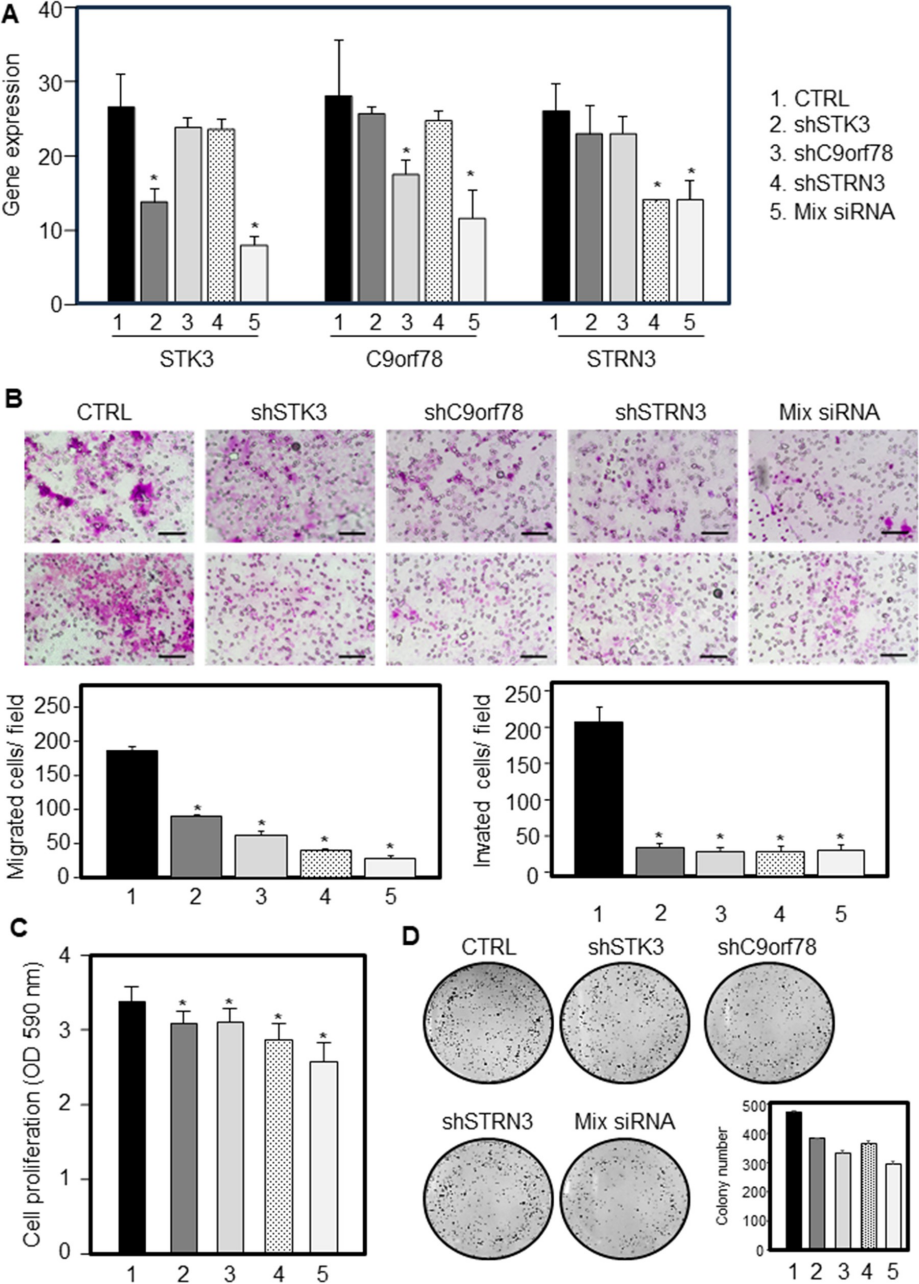
Silencing STK3, C9orf78, STRN3 in NSSCC cells. NSSCC cells were silenced for STK3 (shSTK3), C9orf78 (shC9orf78) and STRN3 (shSTRN3) or for all genes (mix siRNA) and their expression evaluated (**A**). The silenced NSSCC cells and their parental counterparts were investigated for cell migration/invasion (**B**), cell proliferation (**C**) and colony forming assay (**D**). Scale bar = 100 μm. Comparisons among groups were determined by analysis of variance (ANOVA) followed by post-hoc Tukey analysis. Differences with p < 0.05 were considered statistically significant.

The STK3, C9orf78 and STRN3 silencing model was successfully established in NSSCC cells, and the expression of *STK3*, *C9orf78 and STRN3* genes was verified using qRT-PCR (**Fig 7A**). Silencing of *STK3*, *C9orf78 and STRN3* genes markedly inhibited cell migration and invasion (**Fig 7B**), and reduced both cell proliferation (**Fig 7C**) and colony formation (**Fig 7D**). All these effects were amplified when all genes were supressed.

## Discussion

The miRNA/mRNA regulatory transcription of the target genes play important role in cancer tumorigenesis, progression, and response to the therapies [22, 23]. Previously, using NGS-based miRNome assay, the miR-34/miR-449 cluster was identified as the miRNA superfamily involved in the development and progression of SNCs [15, 17]. Differently to miR-34c-5p, which was found highly expressed in oral cancers [24], miR-34c-3p was downregulated in SNCs respect their non-malignant counterparts. Likewise, miR-449 was down expressed in SNC tissues and both miRNAs were differently expressed among the different SNC histotypes. Moreover, the miR-34/miR-449 cluster was an independent prognostic biomarker and was associated with patient outcome [15, 25, 26].

Despite miR-34 and miR-449 sharing the same seed region, they may regulate several different targets. The methods used for miRNA target prediction are technically and analytically difficult [27]. In the present study, we used a sensitive method based on Ago-RIP-Seq after miRNA overexpression to identify the direct targets of the miR-34/miR-449 cluster in SNCs. Targets enriched in Ago-RIP fractions and decreased in total lysate fractions following miR-34/miR-449 overexpression were selected and integrated with ‘in silico’ analysis. Several direct targets have been identified, and most of them were involved in regulation of RNA-DNA metabolic, transcript and epigenetic processes. In particular, *STK3*, *C9orf78* and *STRN3* genes were the common targets of both miR-34c and miR-449a.

Although *STK3*, *C9orf78 and STRN3* genes were post-transcriptionally regulated by miR-34c and miR-449a, their overexpression, rather than inhibiting, induced the expression of STK3, C9orf78 and STRN3. The STK3, C9orf78 and STRN3 overexpression induced cell migration and invasion, which were inhibited when the miR-target genes were silenced, supporting their role in tumorigenicity. Recently has been reported that miRNAs are able not only to repress, but also activate, gene expression, acting on mRNA stability and translation regulation. MiRNAs can bind both the 3’UTR and the 5’UTR of target mRNA, and the association of miRNAs with 5’UTR generally induces an activation of translation rather than a repression [28]. Therefore, we can postulate that the structural miRNA/mRNA interactions could be crucial factors determining different outcomes and biological functions.

By comparing the gene expression of *STK3*, *C9orf78* and *STRN3* in tumour tissue with respect to their non-malignant counterparts, we found that the *STK3*, *C9orf78* and *STRN3* gene were differently expressed among the SNC histotypes, showing low expression in SNADC (ITAC), SNUC and SNEC, while high expression of these genes was found in SNSCC and SNACC. A negative correlation between miR-34/miR-449 and their target genes was found only in the SNACC. All these observations, highlight the heterogenic nature of the tumour and suggest that other mechanisms are involved in the gene regulation. Due to the heterogenicity in miR-target expression among the different histotypes, a representative histotype for each group (low and high miR-target expression) was selected and their prognostic value evaluated. Interestingly, in ITAC with low miR-targets, the lower expression of *STK3*, *C9orf78* and *STRN3* genes was associated with improved overall survival and reduced risk of recurrence. Conversely, in SNSCC with high miR-targets, the lower gene expression of *STK3*, *C9orf78* and *STRN3* predicted worse prognosis.

Low STK3 expression was associated with a worse prognosis in ovarian cancer [29], and its restoration significantly inhibited cell proliferation, apoptosis, and migration in different cancers in vitro and suppressed tumour growth in vivo [30–32]. On the other hand, high expression of STK3 was found in gastric cancer and might exert oncogenic function [33]. Other type of cancers such as prostate cancer and leukaemia showed increased expression of STK3 [34, 35].

Increased expression of STRN was found in different cancers, which induced cell cycle progression and invasiveness by activating downstream transcription factors, including misshapen-like kinase 1, RhoA, TRAF2, NCK-interacting protein kinase and PDGFRA [36]. It has been reported that STRN4 promote cell proliferation and inhibit apoptosis in prostate cancer by regulating the leukaemia/lymphoma-related factor [37]. Likewise, C9orf78 was overexpressed in hepatocellular carcinoma and several other cancer cell lines but not in their normal tissue counterparts, indicating a possible role of this protein in tumorigenesis [38]. However, the molecular mechanism by which this protein works is completely unknown.

Both STK3 and STRN3 are two important components of the Hippo pathway, which is a key tumour suppressor pathway involved in tissue regeneration, angiogenesis, tumour suppression and metastasis, immune response, and drug resistance [39, 40]. The mammalian Hippo pathway consists of a kinase cascade of Mammalian STE20-like 1/2 (STK4, also known as MST1, and STK3, also known as MST2) and Large Tumour Suppressor 1/2 (LATS1/2), which inhibit the primary effectors of the Hippo pathway, Yes Associated Protein (YAP) and WW Domain-containing Transcription Factor (TAZ) [41]. STRN3 as a regulatory subunit of protein phosphatase 2A (PP2A) in STRIPAK [42], can induce MST1/2 dephosphorylation through PP2A and subsequently increase YAP activity [43].

### Conclusions

This study provides the first evidence that miR-34/miR-449 target genes such a *STK3*, *C9orf78 and STRN3* are deregulated in SNC, are involved in tumorigenesis and could be proposed as valuable prognostic biomarkers. However, to confirm the effectiveness of their prognostic value under study, our findings should necessarily request validation through larger perspective and multicentre randomized studies.

## Supporting information

S1 Fig Experimental design of AGO2-RIP-Seq and RNA-Seq to detect miR-34c and miR-449a targets. (TIF)

S2 Fig PCA plots. Distribution of samples on the entire dataset, on the miRNA treatments and on immunoprecipitation.
(TIF)

S3 Fig Differentially expressed genes (DEGs) in miR-34c. DEGs were identified by comparing the mRNA expression in the AGO2-IP, IgG-IP, and input groups against scramble samples DEGs in AGO2-IP miR-34c vs. AGO2-IP scramble corrected for widespread transcriptome secondary change and unspecific RNA binding to sepharose beads were obtained intersecting up-regulated DEGs (Log_2_ Fold Change > 0) of the three comparisons (circled number in the first Venn Diagram). Direct targets were identified by comparing up-regulated DEGs specific of the comparison AGO2-IP miR vs AGO-IP scramble with down-regulated genes (Log_2_ Fold Change < 0) in the comparison between Input miR-34c vs. Input Scramble. Circled number in the second Venn Diagram indicates miR-34c direct targets. The data were from three independent Ago-IP experiments.
(TIF)

S4 Fig Differentially expressed genes (DEGs) in miR-449a. DEGs were identified by comparing the mRNA expression in the AGO2-IP, IgG-IP, and input groups against scramble samples DEGs in AGO2-IP miR-449a vs. AGO2-IP scramble corrected for widespread transcriptome secondary change and unspecific RNA binding to sepharose beads were obtained intersecting up-regulated DEGs (Log_2_ Fold Change > 0) of the three comparisons (circled number in the first Venn Diagram). Direct targets were identified by comparing up-regulated DEGs specific of the comparison AGO2-IP miR vs AGO2-IP scramble with down-regulated genes (Log_2_ Fold Change < 0) in the comparison between Input miR-449a vs. Input Scramble. Circled number in the second Venn Diagram indicates miR-449a direct targets. The data were from three independent Ago-IP experiments.
(TIF)

S5 Fig Gene-gene interaction network. The identified miR target genes were analysed by means of a gene-gene interaction network using functional protein association networks software (String and Cytoscape software). The nodes in the networks are sized by their node degree.
(TIF)

S1 Raw images (PDF)

S1 Table Ratio of exon-mapped reads to total uniquely mapped reads (expression profile efficiency). (PDF)

S2 Table Multivariate Cox regression analysis for overall survival and disease-free survival. (DOCX)
