# Supplementary figures and images for: AGO2-RIP-Seq reveals miR-34/miR-449 cluster targetome in sinonasal cancers
Source: PLoS One. 2024 Jan 12;19(1):e0295997. doi: 10.1371/journal.pone.0295997 (PMC10786392; doi:10.1371/journal.pone.0295997)

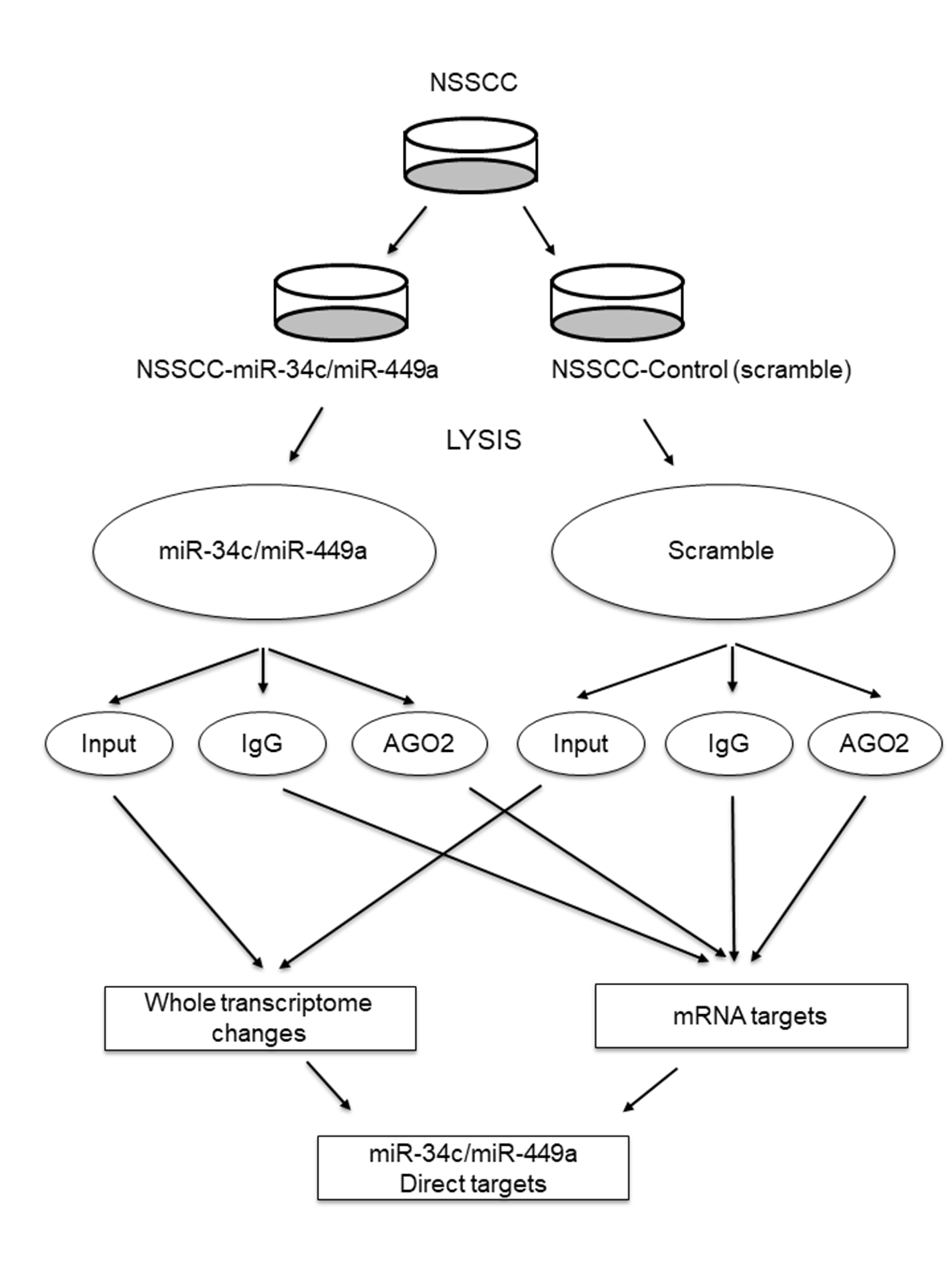

Supplement: S1 Fig — (TIF) [file pone.0295997.s001.tif]

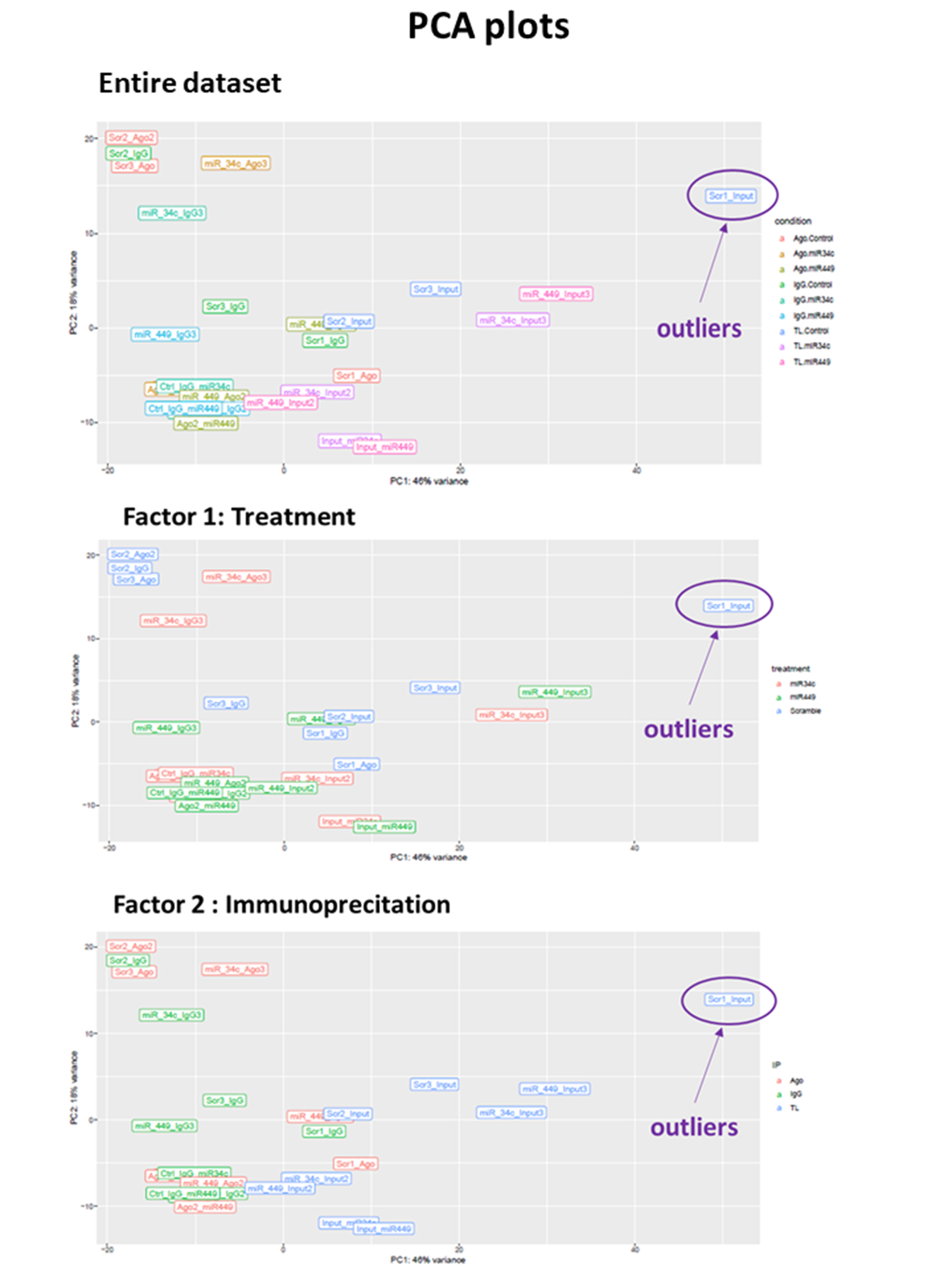

Supplement: S2 Fig — Distribution of samples on the entire dataset, on the miRNA treatments and on immunoprecipitation. (TIF) [file pone.0295997.s002.tif]

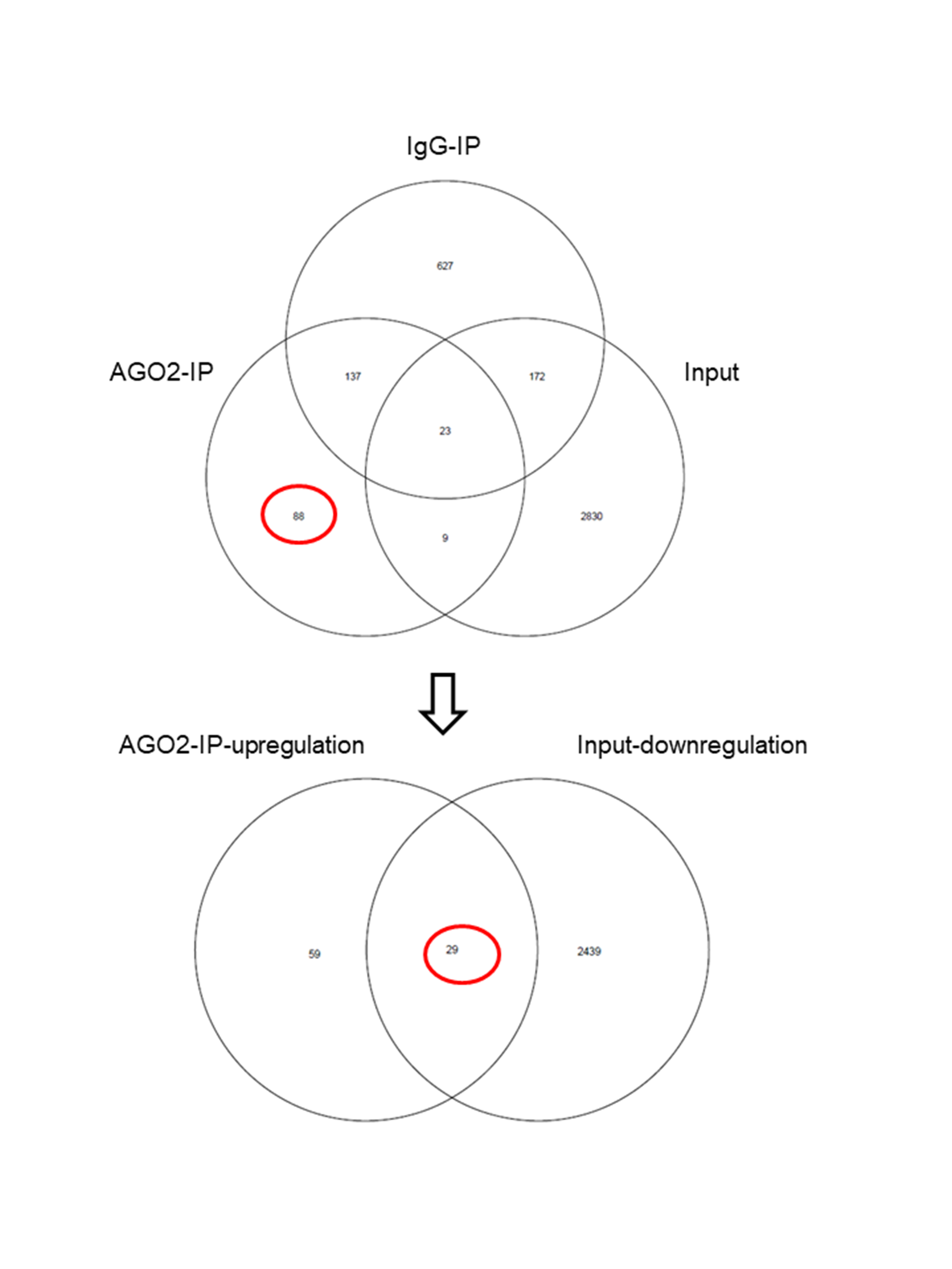

Supplement: S3 Fig — DEGs were identified by comparing the mRNA expression in the AGO2-IP, IgG-IP, and input groups against scramble samples DEGs in AGO2-IP miR-34c vs. AGO2-IP scramble corrected for widespread transcriptome secondary change and unspecific RNA binding to sepharose beads were obtained intersecting up-regulated DEGs (Log2 Fold Change > 0) of the three comparisons (circled number in the first Venn Diagram). Direct targets were identified by comparing up-regulated DEGs specific of the comparison AGO2-IP miR vs AGO-IP scramble with down-regulated genes (Log2 Fold Change < 0) in the comparison between Input miR-34c vs. Input Scramble. Circled number in the second Venn Diagram indicates miR-34c direct targets. The data were from three independent Ago-IP experiments. (TIF) [file pone.0295997.s003.tif]

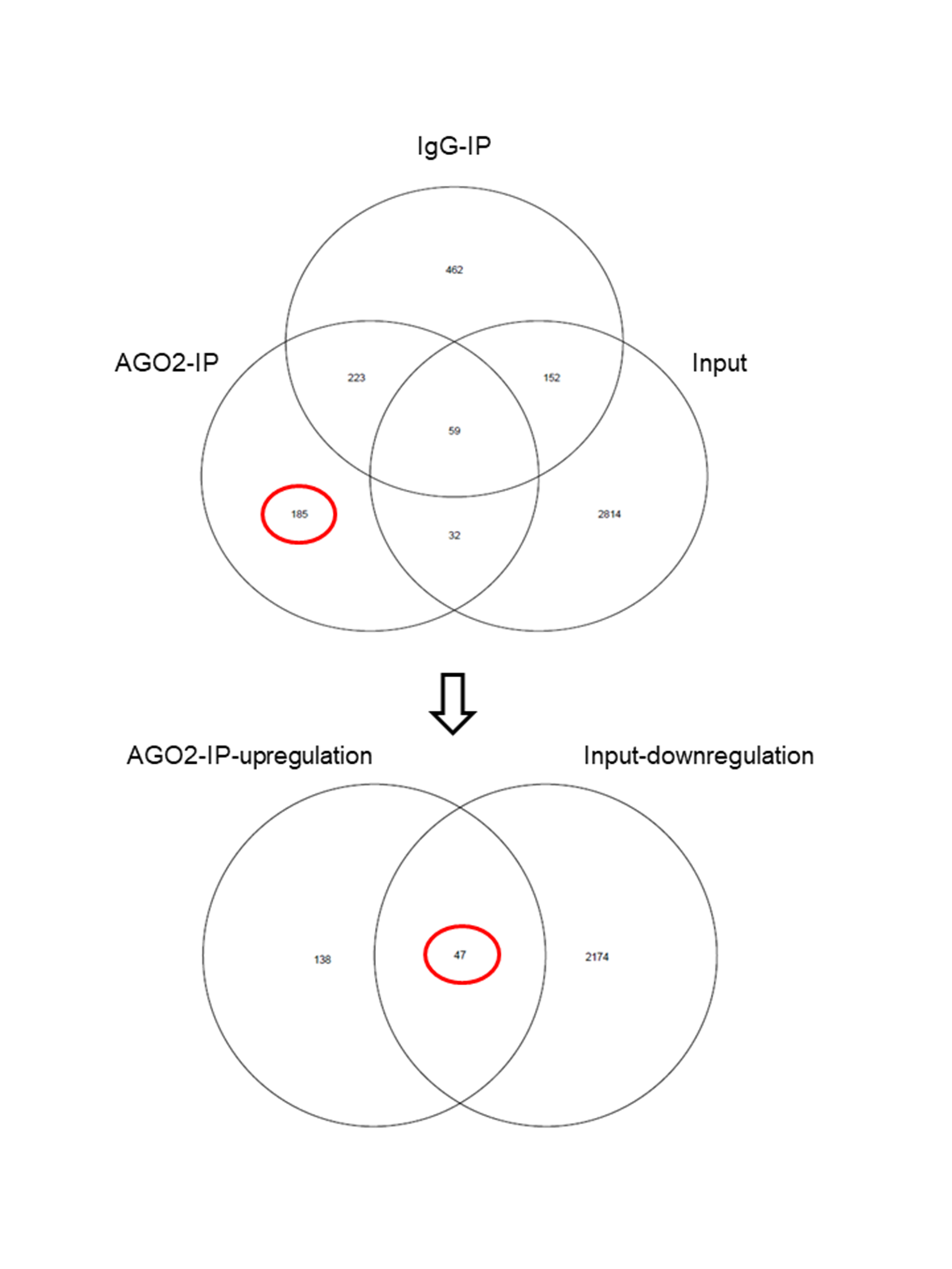

Supplement: S4 Fig — DEGs were identified by comparing the mRNA expression in the AGO2-IP, IgG-IP, and input groups against scramble samples DEGs in AGO2-IP miR-449a vs. AGO2-IP scramble corrected for widespread transcriptome secondary change and unspecific RNA binding to sepharose beads were obtained intersecting up-regulated DEGs (Log2 Fold Change > 0) of the three comparisons (circled number in the first Venn Diagram). Direct targets were identified by comparing up-regulated DEGs specific of the comparison AGO2-IP miR vs AGO2-IP scramble with down-regulated genes (Log2 Fold Change < 0) in the comparison between Input miR-449a vs. Input Scramble. Circled number in the second Venn Diagram indicates miR-449a direct targets. The data were from three independent Ago-IP experiments. (TIF) [file pone.0295997.s004.tif]

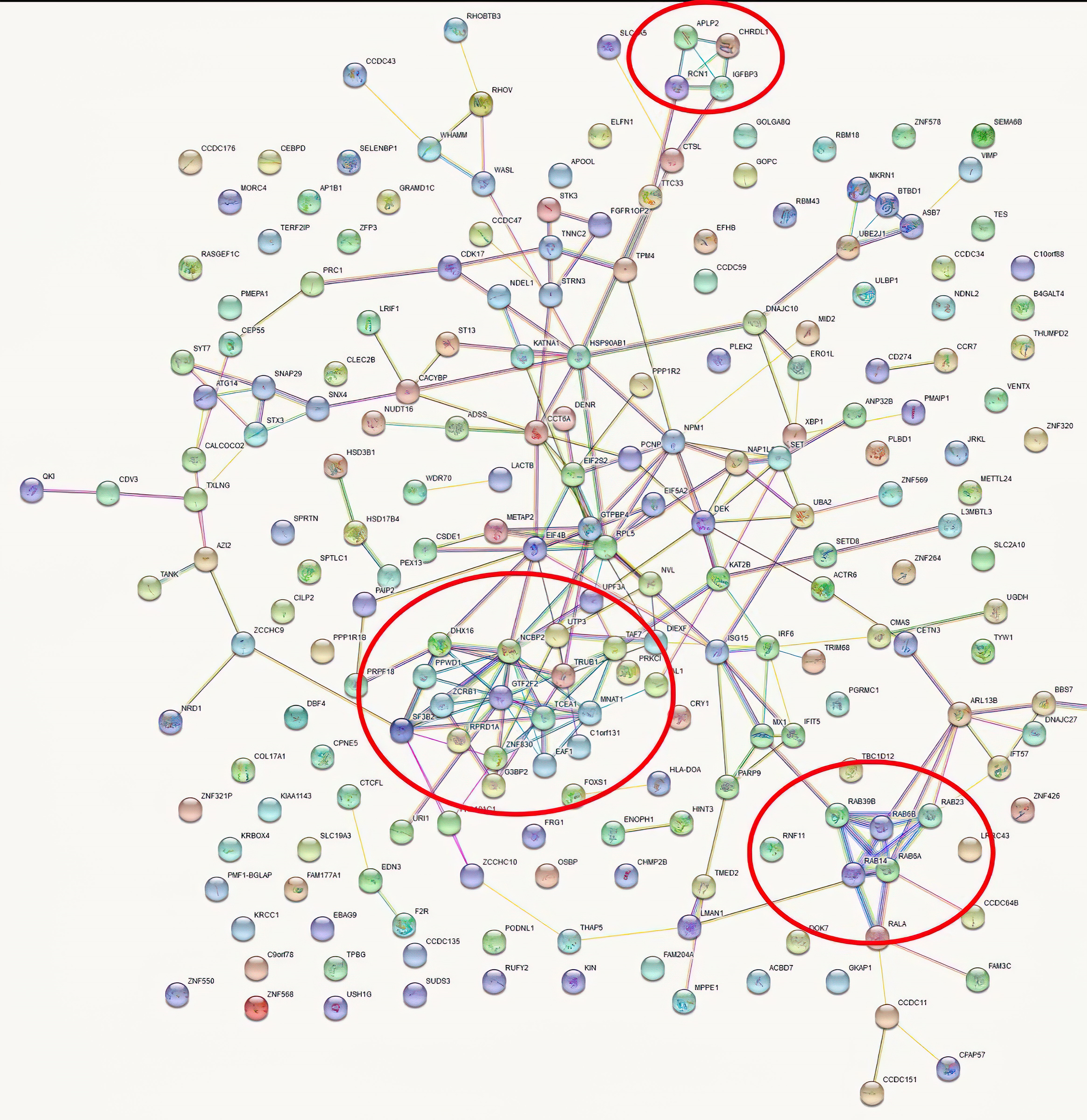

Supplement: S5 Fig — The identified miR target genes were analysed by means of a gene-gene interaction network using functional protein association networks software (String and Cytoscape software). The nodes in the networks are sized by their node degree. (TIF) [file pone.0295997.s005.tif]

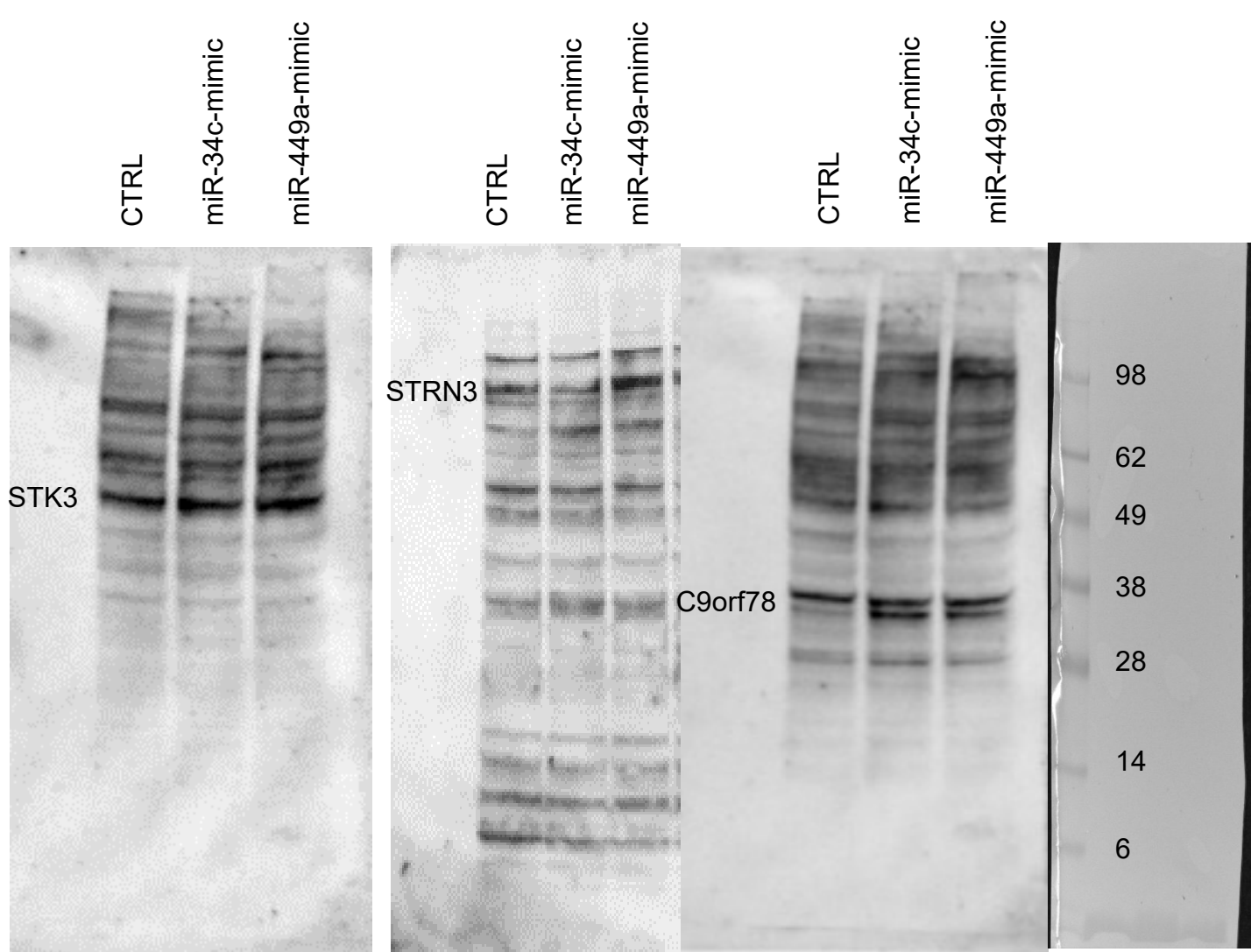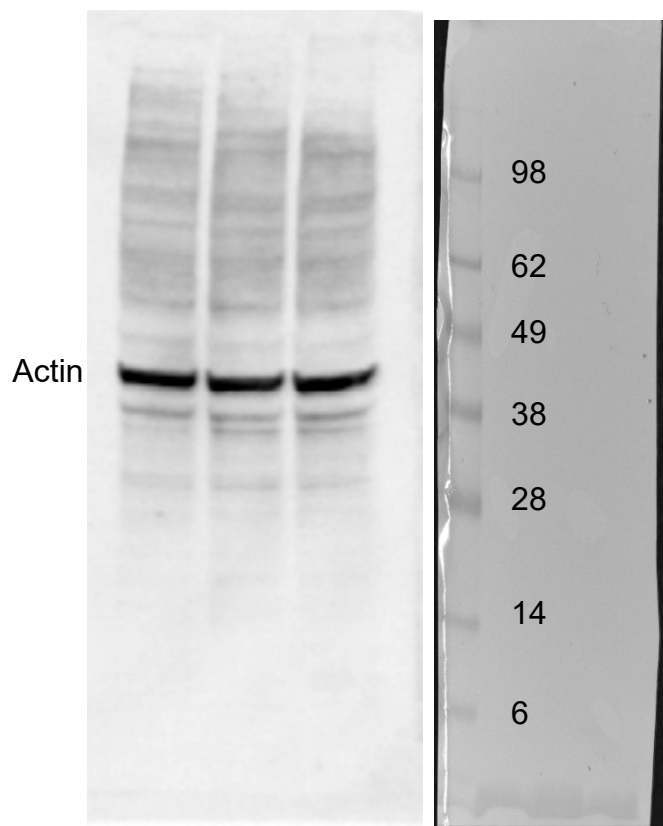

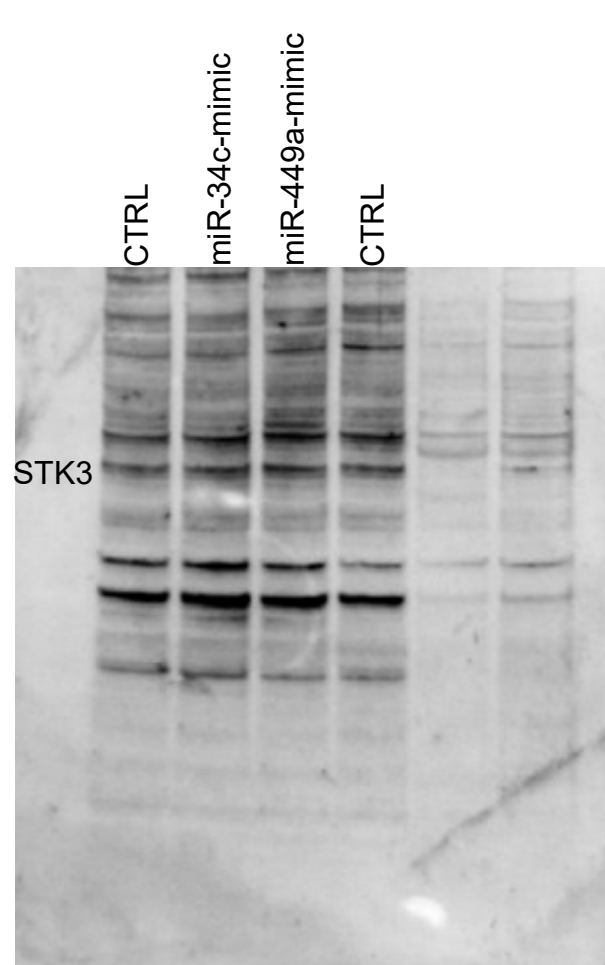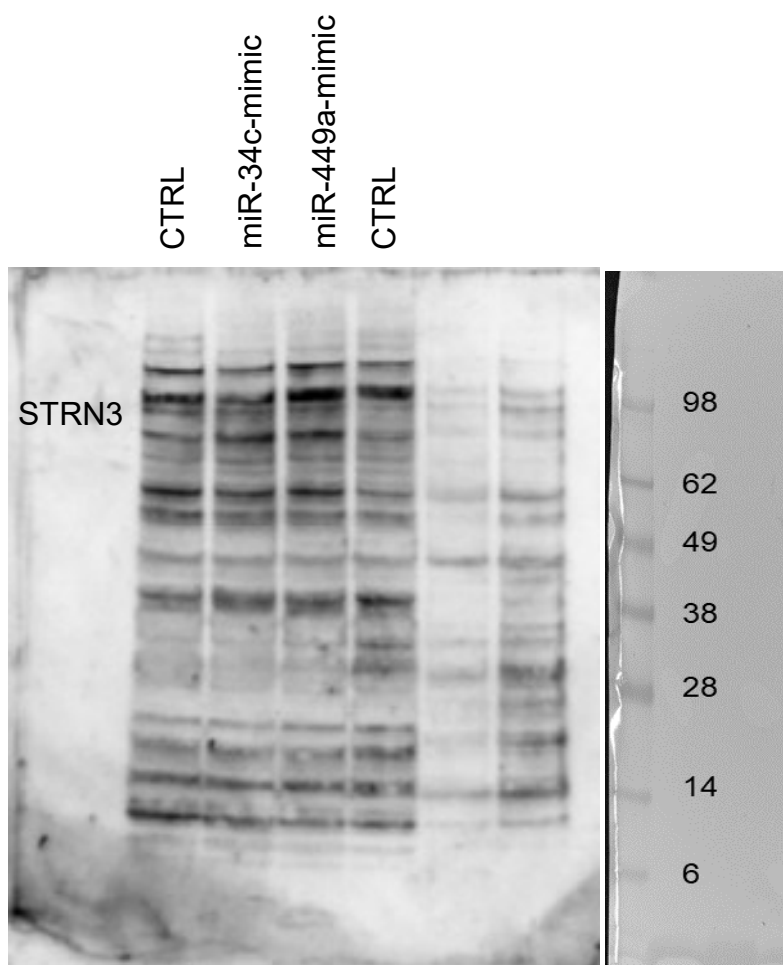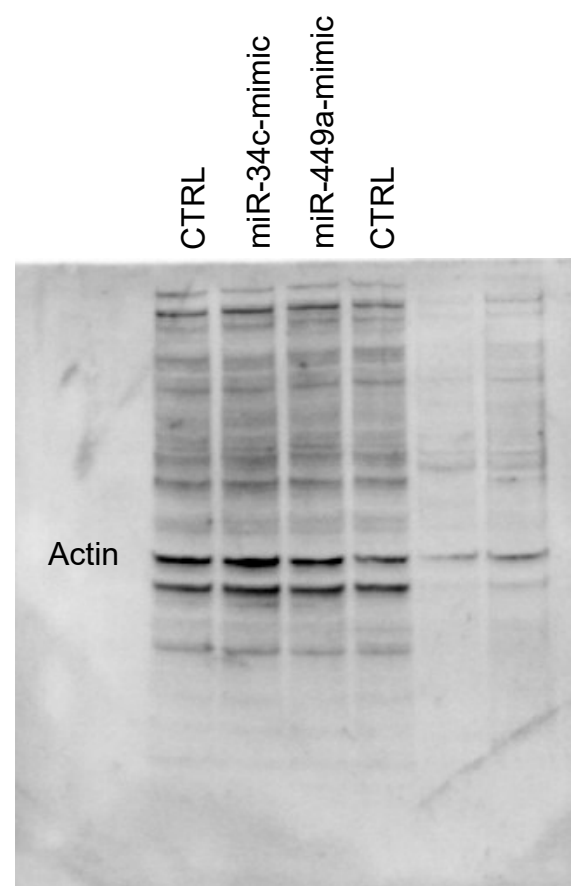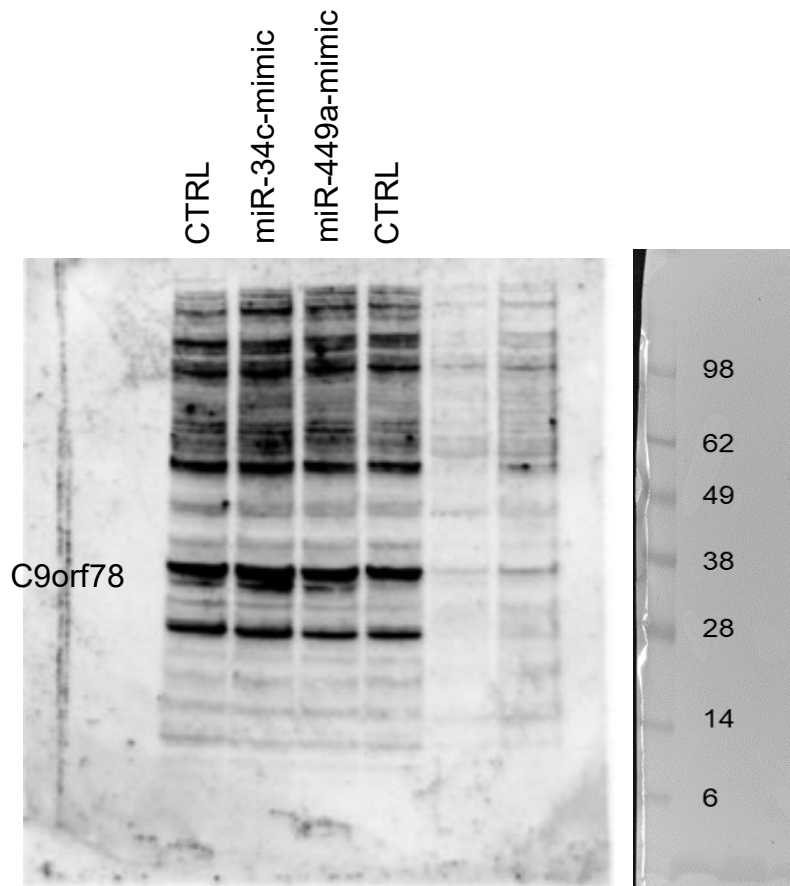

Supplement: S1 Raw images — (PDF) [file pone.0295997.s006.pdf]
