# Supplementary material for: AGO2-RIP-Seq reveals miR-34/miR-449 cluster targetome in sinonasal cancers
Source: PLoS One. 2024 Jan 12;19(1):e0295997. doi: 10.1371/journal.pone.0295997 (PMC10786392; doi:10.1371/journal.pone.0295997)
Supplement: S1 Table — (PDF) [file pone.0295997.s007.pdf]

Sample	Sequenced reads	Reads trimmed	Mapped reads	mirna with count ≥ 1	mirna with count ≥ 3
**SNC-ITAC 1P**	7059152	2409915	1783081	1098	655
**SNC-ITAC 1S**	6831069	3321659	2928065	1088	672
**SNC-ITAC 2P**	7084636	4511576	4130861	1335	742
**SNC-ITAC 2S**	5745407	3230613	2842623	1196	685
**SNC-ITAC 3P**	8338137	1021359	163913	863	443
**SNC-ITAC 3S**	7273658	1657649	297816	701	376
**SNC-ITAC 4P**	8016539	1710809	310142	826	427
**SNC-ITAC 4S**	6992445	3579095	3079571	1126	656
**SNC-ITAC 5P**	9269080	4124175	2014801	1801	921
**SNC-ITAC 5S**	7282212	2610087	1571648	1349	697
**SNC-SCC 2P**	9939564	2447158	1049558	1362	718
**SNC-SCC 2S**	9039470	4813944	3791870	1523	910
**SNC-SCC 3P**	9828667	5902170	4774886	1450	851
**SNC-SCC 3S**	10533703	5560391	4427301	1516	926
**SNC-SCC 4S**	9247437	4706273	3637045	1556	904
**SNC-SCC 1P**	8029693	2287189	1524943	1304	750
**SNC-SCC 1S**	7605071	3914344	2850195	1259	723
**SNC-SCC 4P**	7869415	4004622	3472028	1271	729
**SNC-SCC 5S**	8291390	3140354	2132109	1199	665
