# Supplementary material for: AGO2-RIP-Seq reveals miR-34/miR-449 cluster targetome in sinonasal cancers
Source: PLoS One. 2024 Jan 12;19(1):e0295997. doi: 10.1371/journal.pone.0295997 (PMC10786392; doi:10.1371/journal.pone.0295997)
Supplement: S2 Table — (DOCX) [file pone.0295997.s008.docx]

**Table S2**. Multivariate Cox regression analysis for overall survival and disease-free survival in ITAC

| **OS** | **HR** | **95% CI (HR)** | **p-value** |
| --- | --- | --- | --- |
| **Age** | 0.93 | 0.86-1.02 | 0.126 |
| **Smoking**  No  Yes  Former | 0.65  0.43 | 0.08-5.61  0.04-4.16 | 0.764  0.700  0.463 |
| **TNM classification**  S1/S2  **S3/S4** | **32.20** | **1.65-626.32** | **0.022** |
| **Recurrence** | **0.02** | **0.01-0.38** | **0.009** |
| **STK3 (**low vs high) | 29.96 | 0.24-3.8x10^3^ | 0.169 |
| **C9orf78 (**low vs high) | 0.28 | 0.01-5.62 | 0.407 |
| **STRN3** (low vs high) | **0.001** | **0.001-0.12** | **0.018** |
|  |  |  |  |
| **DFS** | **HR** | **95% CI (HR)** | **p-value** |
| **Age** | 1.00 | 0.94-1.08 | 0.90 |
| **Smoking**  No  Yes  Former | 0.25  0.95 | 0.03-1.90  0.16-5.53 | 0.291  0.180  0.956 |
| **TNM classification**  S1/S2  S3/S4 | 0.29 | 0.05-1.59 | 0.152 |
| **STK3 (**low vs high) | 0.38 | 0.03-4.91 | 0.460 |
| **C9orf78 (**low vs high) | 2.16 | 0.82-5.71 | 0.122 |
| **STRN3** (low vs high) | **1.42** | **1.05-1.93** | **0.024** |
|  |  |  |  |

Regression model with stepwise Wald-backward adjusted for age, gender, smoking, histotype, TNM classification, recurrence, and miRNAs. HR, hazard ratio; CI, confidence interval.
